# Mapping quantitative trait loci associated with self-(in)compatibility in goji berries (*Lycium barbarum*)

**DOI:** 10.1186/s12870-024-05092-7

**Published:** 2024-05-23

**Authors:** Cuiping Wang, Ken Qin, Xiaohui Shang, Yan Gao, Jiali Wu, Haijun Ma, Zhaojun Wei, Guoli Dai

**Affiliations:** 1 School of Biological Science and Engineering, North Minzu University, Yinchuan, 750021 China; 2 State Key Laboratory of Efficient Production of Forest Resources, Yinchuan, 750004 China; 3 National Wolfberry Engineering Research Center, Ningxia Academy of Agriculture and Forestry Sciences, Yinchuan, 750002 China; 4 Ningxia Grape and Wine Technology Center, North Minzu University, Yinchuan, 750021 China

**Keywords:** *Lycium barbarum*, Genetic map, Self-incompatibility, Quantitative trait loci, S-factor

## Abstract

**Background:**

Goji (*Lycium barbarum* L.) is a perennial deciduous shrub widely distributed in arid and semiarid regions of Northwest China. It is highly valued for its medicinal and functional properties. Most goji varieties are naturally self-incompatible, posing challenges in breeding and cultivation. Self-incompatibility is a complex genetic trait, with ongoing debates regarding the number of self-incompatible loci. To date, no genetic mappings has been conducted for *S* loci or other loci related to self-incompatibility in goji.

**Results:**

We used genome resequencing to create a high-resolution map for detecting de novo single-nucleotide polymorphisms (SNP) in goji. We focused on 229 F1 individuals from self-compatible ‘13–19’ and self-incompatible ‘new 9’ varieties. Subsequently, we conducted a quantitative trait locus (QTL) analysis on traits associated with self-compatibility in goji berries. The genetic map consisted of 249,327 SNPs distributed across 12 linkage groups (LGs), spanning a total distance of 1243.74 cM, with an average interval of 0.002 cM. Phenotypic data related to self-incompatibility, such as average fruit weight, fruit rate, compatibility index, and comparable compatibility index after self-pollination and geitonogamy, were collected for the years 2021–2022, as well as for an extra year representing the mean data from 2021 to 2022 (2021/22). A total of 43 significant QTL, corresponding to multiple traits were identified, accounting for more than 11% of the observed phenotypic variation. Notably, a specific QTL on chromosome 2 consistently appeared across different years, irrespective of the relationship between self-pollination and geitonogamy. Within the localization interval, 1180 genes were annotated, including Lba02g01102 (annotated as an *S-RNase* gene), which showed pistil-specific expression. Cloning of *S-RNase* genes revealed that the parents had two different *S-RNase* alleles, namely *S1S11* and *S2S8*. S-genotype identification of the F1 population indicated segregation of the four S-alleles from the parents in the offspring, with the type of *S-RNase* gene significantly associated with self-compatibility.

**Conclusions:**

In summary, our study provides valuable insights into the genetic mechanism underlying self-compatibility in goji berries. This highlights the importance of further positional cloning investigations and emphasizes the importance of integration of marker-assisted selection in goji breeding programs.

**Supplementary Information:**

The online version contains supplementary material available at 10.1186/s12870-024-05092-7.

## Background

Goji (*Lycium barbarum* L.), a perennial shrub with deciduous fruit that belongs to the Solanaceae family. It is widely distributed throughout China, Europe, the United States of America, and the Mediterranean region. For centuries, the fruit of goji, commonly known as goji, has been used in traditional Chinese herbal medicine and as a nourishing tonic [1]. Goji berries have numerous health benefits, including vision enhancement, cancer cell growth inhibition, fatigue reduction, anti-aging effects, metabolism enhancement, and immune system strengthening [2]. These impressive effects can be attributed to the presence of several key active compounds in goji berries, including alkaloids, flavonoids, glycosides, pigments, organic acids, and polysaccharides [3].

Self-incompatibility (SI) is a common mechanism observed in flowering plants to facilitate self-pollen identification and rejection, thus hindering self-fertilization [4]. An incompatible reaction occurs when the stigma and pollen possess the same S-haplotype [5]. The presence of SI in plant populations encourages outcrossing, which leads to increased levels of heterozygosity and genetic diversity, in turn, enhancing resilience and facilitating the adaptation to environmental changes.

However, the presence of a functional SI system in economic forest crop species poses challenges for crop cultivators [6]. In particular, the presence of SI in goji plants poses challenges when cultivating single varieties in large areas, resulting in large flower and fruit drops, inconsistent fruit size, very low seed-setting rates, and even loss of basic harvest. To address this challenge, interplanting pollinated trees becomes necessary during cultivation, which not only increases the production complexity but also limits the promotion and application of new varieties. Although varieties such as ‘*Ningqi* 1’ and ‘*Ningqi* 7’ are widely planted in Northwest China and are self-compatible, other excellent varieties such as ‘*Ningqi* 6’ and ‘*Ningqi* 8’ are self-incompatible and have not been widely promoted. Therefore, self-compatibility has become the most crucial factor in goji breeding programs in recent years.

In the context of SI, the ability of pollen to be accepted by the pistil is determined by its genetic background. SI can be classified into two types based on the phenotype of pollen incompatibility and the mode of genetic control: Gametophytic self-incompatibility (GSI) and sporophytic self-incompatibility (SSI). The GSI group includes Solanaceae, Rosaceae, Rubiaceae, and Papaveraceae, where the compatibility of pollen with the pistil is determined by the genotype of the pollen (gametophyte). In SSI, the SI phenotype of pollen is determined by the genotype of the diploid parent (sporophyte) [7]. The SI in Brassicaceae belongs to the SSI group. The S-determining factor of its pistil, known as the S-locus receptor kinase (SRK), was the first to be identified [8]. The stamen S-determining factor is the S-locus cysteine-rich protein (SCR), also referred to as S-locus protein 11 (SP11) [9]. In Papaveraceae, SI is a GSI, and the *S* genes for both the pistil and stamen have been identified. The pistil *S* gene encodes a 15 kDa secreted protein called *Papaver rhoeas* stigma S (PrsS), while the stamen *S* gene encodes a 20 kDa transmembrane protein known as *P. rhoeas* pollen S (PrpS) [10]. Additionally, there is another type of SI mechanism regulated by a distinct pair of pistil and stamen S genes, which is found in Solanaceae, Rosaceae, and Rubiaceae. SI in solanaceous plants is a GSI system that relies on the *S*-locus mechanism. The female S-determinant is encoded by a group of glycoproteins with ribonuclease activity known as S-ribonucleases (S-RNases) [11, 12], while the pollen S-determinant is encoded by a class of genes with an F-box structure, collectively known as S-locus F-box (*SLF*) genes [13]. However, SI is genetically complex, quantitative in nature, and controlled by an unknown number of loci. Studies by Kakita et al. (2007) and Ma et al. (2021) demonstrated cases of self-compatibility that cannot be explained by known *S*-loci, suggesting the involvement of new loci in determining self-compatibility [14, 15]. Calcium (Ca^2+^) signaling has been identified as a crucial factor in the detection and/or suppression of self-pollen in SI mechanisms [16]. Partial alleviation of SI can be achieved by administering chemical reagents that modulate Ca^2+^ channeling across cell membranes to self-pollinated stigmas [17].

The process of SI in goji involves recognition between pollen and self-pistils, which is mediated by multiple alleles of the *S* locus. These alleles are closely related at the locus and function as a complete genetic unit. In self-incompatible goji plants, pollen can germinate on the stigma and initiate tube elongation. However, growth inhibition of the pollen tube occurs in the conducting tissue of the style, which serves as direct evidence that the SI system in goji is gametophytic.

Quantitative mapping of trait loci (QTLs) is a valuable approach for the identification of functional loci and genes. Creating a high-quality genetic link map is vital for QTL mapping, as it forms the basis for subsequent analyses and identification of trait-associated loci. QTL mapping has also been used to investigate SI loci in various plant species. For example, in 1997, QTLs associated with the *S* locus and several crucial floral traits related to pollination biology were mapped using a BC1 population derived from the self-compatible tomato species *Lycopersicon esculentum* and the self-incompatible wild species *L. hirsutum f. typicum* [18]. Similarly, in sunflower (*Helianthus annuus* L.), the QTLs for SI were genetically mapped using a backcross population created from an elite, self-pollinated, nondormant inbred line (NMS373) and a wild, self-incompatible, dormant population (ANN1811) [19]. Furthermore, a series of QTLs associated with SI were successfully mapped in perennial ryegrass (*Lolium perenne*) [20–24]. Additionally, two QTLs controlling self-compatibility were identified in *Brassica rapa*.

In the case of goji plants, QTL mapping analysis has recently gained attention, focusing on fruit yield traits. Using double digest restriction site-associated DNA sequencing (ddRAD-seq) on an intraspecific F1 population, Gong et al. (2019) created the first high-density genetic map of goji, comprising 23,967 SNPs, with a total genetic length of 964.03 cM and an average interval of 0.040 cM. Through QTL analysis of a two-year dataset, researchers identified eight critical loci associated with photosynthetic traits [25]. Additionally, Zhao et al. (2019) described the construction of a genetic map for goji using specific locus amplified fragment (SLAF) sequencing. This SNP-based genetic map consisted of 6,733 SNPs spanning a total length of 1,702.45 cM, with an average intermarker distance of 0.31 cM. Through QTL analysis of two- and three-year datasets, researchers identified a total of 55 QTLs, including 18 QTLs for the fruit-related phenotype on linkage group (LG) 11 and two crucial QTLs for the leaf index on different LGs [26]. Furthermore, Rehman et al. (2020) constructed a high-resolution map of *L. chinense* and *L. barbarum* using SLAF sequencing, which comprised 3,495 SLAF markers in 12 LGs, spanning 1,649.03 cM with an average interval of 0.47 cM. A total of 117 QTLs related to multiple traits associated with fruit were detected, including 78 QTLs in two individual years and 36 QTLs in an additional year [27]. Zhao et al. (2021) reported a high-density genetic map using SNPs determined by whole-genome resequencing, which contained 8,507 SNPs, covering a genetic distance of 2,122.24 cM with an average distance of 0.25 cM based on an F1 population. Researchers have identified 25 stable QTLs for the associated characteristics of leaves and fruits [28]. Furthermore, Yin (2022) constructed a genetic map using a combination of 74 amplified fragment length polymorphism (AFLP) markers and 91 simple sequence repeat (SSR) markers, which were distributed across 12 linkage groups. The total genetic length of the map was 557.6 cM, with an average intermarker distance of 3.38 cM [29].

The Ningxia Hui Autonomous Region is widely regarded as the “*daodi*” goji production area in China, due to its unique geographical location and favorable growth conditions. This region has established itself as the main location for goji cultivation and is characterized by stable growth, exceptional quality, and efficient production [30]. By 2021, the goji planting area in Ningxia covered 30,000 hectares, resulting in an impressive output of 300,000 tons.

In this study, we used a population of 229 F1 individuals derived from inner-specific crosses between the self-compatible cultivar ‘13–19’ and the SI cultivar ‘new 9’ (both belonging to *L. barbarum*). Our objectives were to locate QTLs linked to self-compatible loci in *L. barbarum*, and to classify potential candidate factors that control the transition of SI in *L. barbarum*. These findings aim to provide valuable information on self-compatible parents and loci for future molecular breeding efforts in goji.

## Results

### Variability of morphological traits

**Fig. 1:**
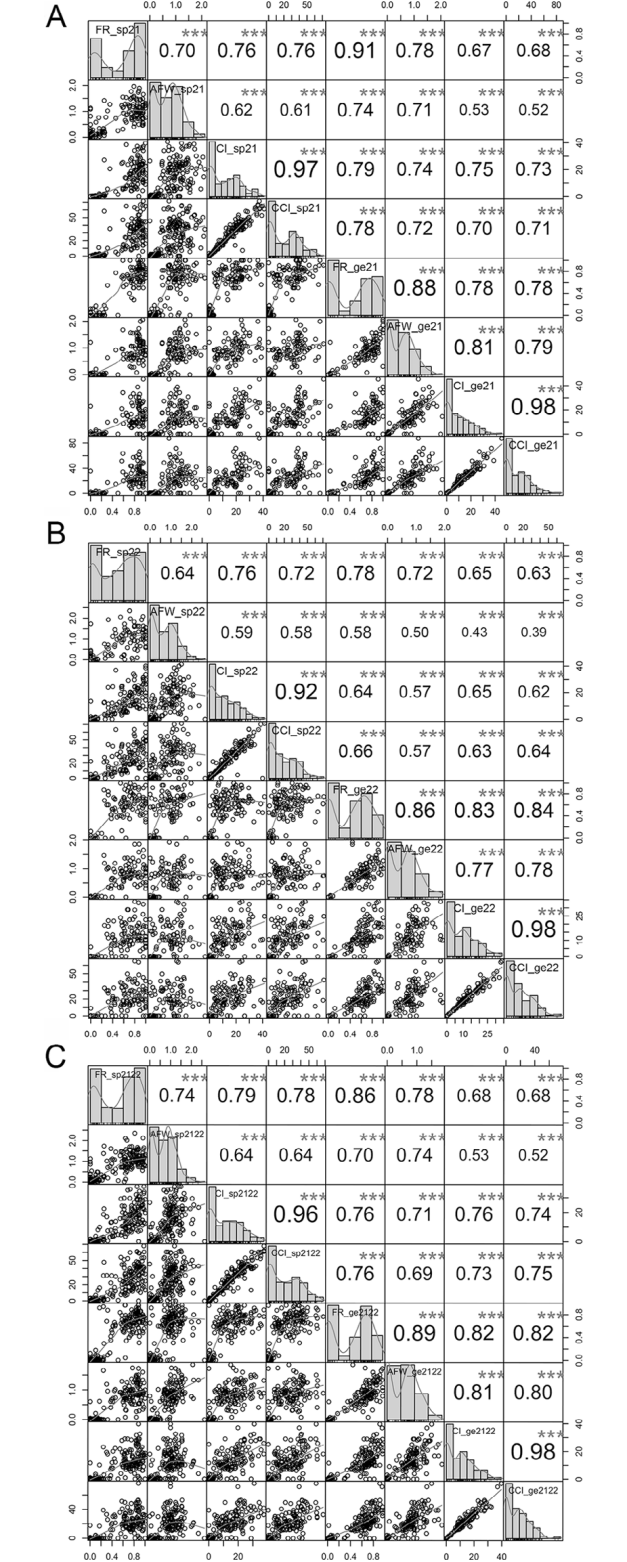
Frequency distribution of four self-incompatibility (SI)-related traits for the F1 population after self-pollination (_sp) **(A)** and geitonogamy (_ge) **(B)** in 2021 and 2022; the additional year represents the mean 2021/22, respectively. Histograms are displayed for compatibility index (CI), compared compatibility index (CCI), fruitful rate (FR), and average fruit weight (AFW). The x-axis of the plots represents the value of the four traits, while the y-axis indicates the number of trees that exhibits the corresponding value on the x-axis

The SI-related agronomic traits (FR, AFW, CI, and CCI) of the F1 population were evaluated after artificial self-pollination and geitonogamy from 2021 to 2022, as well as an extra year representing the mean data from 2021 to 2022 (2021/22). Analysis of variance revealed no statistically significant differences (*P* < 0.05 or *P* < 0.01) in morphological traits between the F1 offspring over different years (Supplementary Table S1), suggesting the presence of great variation and the resilience of these traits to environmental influences. Descriptive statistical analysis revealed that the coefficient of variation changed for different traits and years. For example, the coefficient of variation ranged from 65% for FR after self-pollination in 2021 to 105% for CCI after geitonogamy in 2021 (Table 1). The frequency distribution histogram and box plot for each year (2021 and 2022) and the additional year (2021/22) revealed nonnormal distributions among all morphological traits. The SI-related traits exhibited an approximately exponential distribution, indicating a combination of exponential and normal distributions (Fig. 1). For both self-pollination and geitonogamy, all four detection indicators exhibited a high distribution frequency when the value was 0 or close to 0. Pearson’s correlation analysis indicated a highly significant positive association (*P* < 0.001) between CI, CCI, FR, and AFW (Fig. 1). The correlation between CI and CCI is stronger under the same pollination method, with a correlation index exceeding 0.92. Both self-pollination and geitonogamy within the same plant were significantly correlated with the same indicator, with a greater correlation coefficient for FR, which was 0.78 in 2021 and 0.91 in 2022 (Fig. 1).

**Table 1:** Descriptive statistics of eight attributes based on datasets of two individual years (2021–2022) and an extra year (2021/22)

Traits	Years	Mean ± SD	SE	Maximum	Minimum	Skewness	Kurtosis	Variance	CV %
FR_sp21	2021	0.57 ± 0.39	0.03	1.00	0.00	-0.48	-1.48	0.14	0.65
FR_sp22	2022	0.51 ± 0.35	0.03	1.00	0.00	-0.23	-1.36	0.12	0.68
FR_sp21/22	2021/22	0.52 ± 0.35	0.02	1.00	0.00	-0.33	-1.48	0.12	0.67
AFW_sp21	2021	0.82 ± 0.56	0.04	2.12	0.00	0.14	-0.89	0.31	0.68
AFW_sp22	2022	0.76 ± 0.64	0.05	2.64	0.00	0.41	-0.74	0.41	0.84
AFW_sp21/22	2021/22	0.80 ± 0.58	0.04	2.64	0.00	0.28	-0.51	0.33	0.72
CI_sp21	2021	12.91 ± 11.74	0.81	40.80	0.00	0.45	-0.96	137.75	0.91
CI_sp22	2022	11.64 ± 10.61	0.77	41.42	0.00	0.67	-0.48	112.51	0.91
CI_sp21/22	2021/22	12.18 ± 10.79	0.73	37.41	0.00	0.48	-0.88	116.44	0.89
CCI_sp21	2021	22.32 ± 20.24	1.39	71.54	0.00	0.41	-1.08	409.79	0.91
CCI_sp22	2022	20.09 ± 18.34	1.33	71.17	0.00	0.64	-0.53	336.48	0.91
CCI_sp21/22	2021/22	21.07 ± 18.59	1.24	68.01	0.00	0.45	-0.98	345.55	0.88
FR_ge21	2021	0.47 ± 0.37	0.03	1.00	0.00	-0.23	-1.62	0.14	0.78
FR_ge22	2022	0.44 ± 0.34	0.03	1.00	0.00	-0.20	-1.40	0.11	0.76
FR_ge21/22	2021/22	0.46 ± 0.34	0.02	0.96	0.00	-0.32	-1.51	0.11	0.74
AFW_ge21	2021	0.61 ± 0.55	0.04	2.06	0.00	0.38	-0.83	0.30	0.89
AFW_ge22	2022	0.60 ± 0.51	0.04	1.93	0.00	0.38	-0.62	0.26	0.86
AFW_ge21/22	2021/22	0.61 ± 0.50	0.04	1.85	0.00	0.18	-1.04	0.25	0.83
CI_ge21	2021	10.32 ± 10.77	0.90	45.44	0.00	0.85	-0.03	115.91	1.04
CI_ge22	2022	9.46 ± 9.06	0.73	33.91	0.00	0.66	-0.43	82.15	0.96
CI_ge21/22	2021/22	9.85 ± 9.42	0.69	39.67	0.00	0.70	-0.22	88.68	0.96
CCI_ge21	2021	18.43 ± 19.29	1.61	87.38	0.00	0.923	0.401	372.24	1.05
CCI_ge22	2022	16.13 ± 15.72	1.25	65.21	0.00	0.699	-0.217	247.02	0.97
CCI_ge21/22	2021/22	17.15 ± 16.46	1.20	76.29	0.00	0.774	0.217	270.96	0.96

FR, fruitful rate; AFW, average fruit weight; CI, self-compatibility index; CCI, compared self-compatibility index; _sp, collected after self-pollination; _ge, collected after geitonogamy

### Genome resequencing and genotyping

**Fig. 2:**
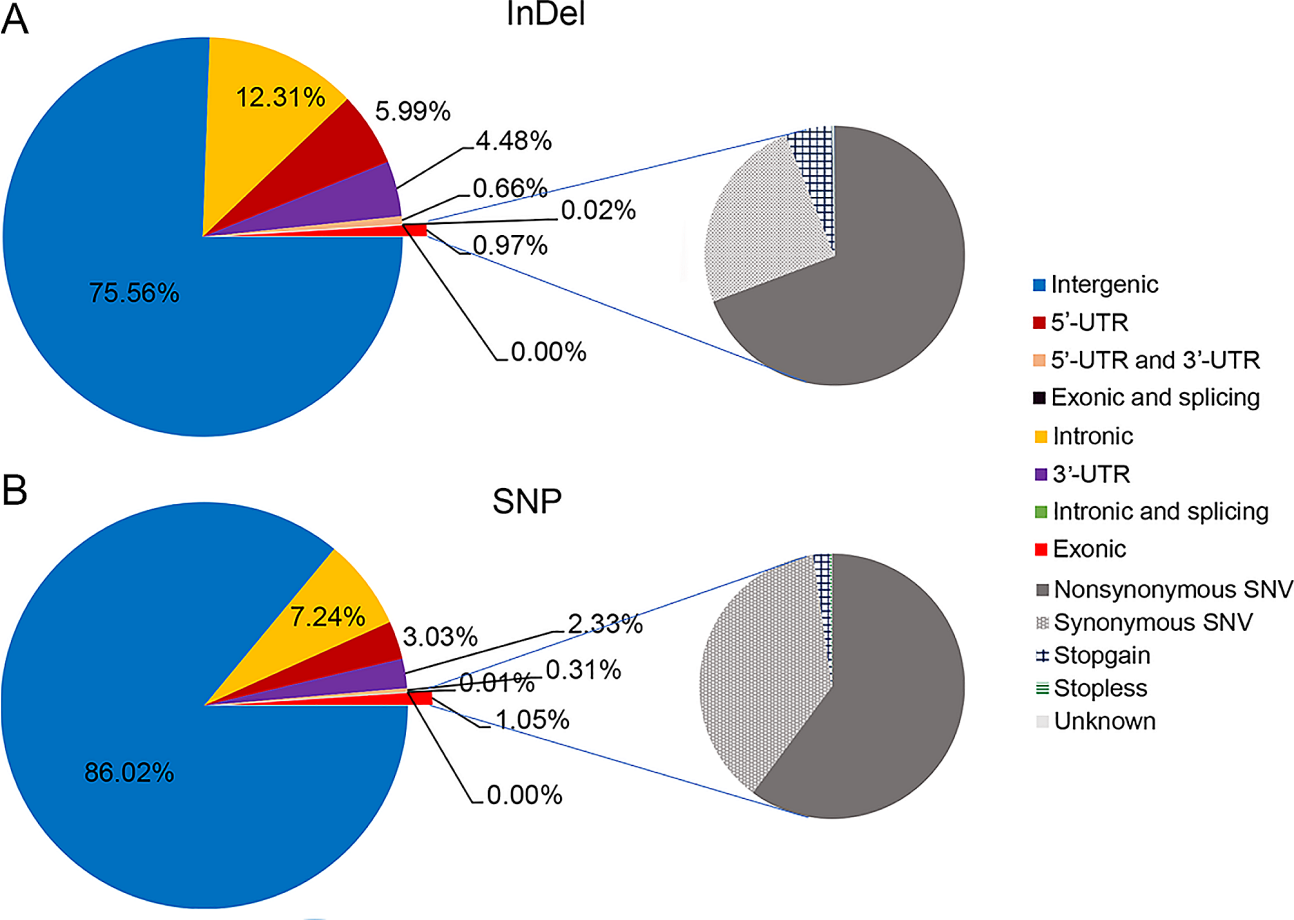
Percentage and type of single-nucleotide polymorphisms (SNPs) and insertion-deletions (InDels) obtained by genome resequencing. **(A)** Left: Percentage plot of InDel distribution; right: Pie charts of InDel annotation information. **(B)** Left: Percentage plot of SNP distribution; right: Pie charts of SNP annotation information. SNV: single nucleotide variation

After removing adapters and low-quality reads, a total of 2354 Gb of raw reads were generated, resulting in 2325 Gb of high-quality clean reads derived from the 229 individuals in the F1 population and the parents. The average percentage of clean reads in the genome was 92.12%, with an average mapping rate of 99.71% (Supplementary Table S2). Based on the alignment results to the reference genome, a total of 33,603,135 SNPs were identified, including 20,999,363 transitions and 12,603,772 transversions. Most of the annotated SNPs (63.74%) were found in intergenic regions, indicating that they are outside of the coding sequences of the genes, while 18% of the SNPs were identified within the coding sequence (CDS) region, suggesting potential functional effects on the protein product. Furthermore, a significant portion of the SNPs (54.66%) were nonsynonymous, resulting in an amino acid change in the protein sequence (Fig. 2). Additionally, 6,288,815 small InDels were detected, including 3,237,526 deletions and 3,051,289 insertions. Similar to SNPs, most InDels (53.87%) were annotated in intergenic regions, indicating their presence outside CDSs. Only a small proportion (1.37%) of InDels were found in the CDS region. Among InDels in the CDS, a large proportion (60.77%) led to frameshift mutations, which can have a significant impact on the resulting protein product (Fig. 2; Supplementary Table S3). In terms of SNP classification, a total of 8,734,495 SNPs were successfully assigned to eight genotyping patterns, providing insight into the genetic variations. A subset of 8,999,876 SNPs was used to develop a high-density genetic map of goji.

### Genetic map construction

To ensure the accuracy and reliability of the genetic map, certain filtering criteria were applied. Markers with integrity ≤ 70% were excluded, as were highly significant SNPs with a significant standard deviation (SD) value of *P* < 0.0001. In the case of the F1 cross-pollinated population, seven specific segregation patterns (ab × cd, ef × e.g., hk × hk, lm × ll, nn × np, ab × cc, and cc × ab) were identified and selected for genetic mapping to represent the inheritance patterns and provide valuable information for constructing the genetic map. A total of 249,327 SNPs were selected to construct a high-density genetic map of the goji berry. The average sequencing depth for both parental and offspring individuals exceeded 3-fold, ensuring robust and comprehensive coverage of the genetic variations in the population.

**Fig. 3:**
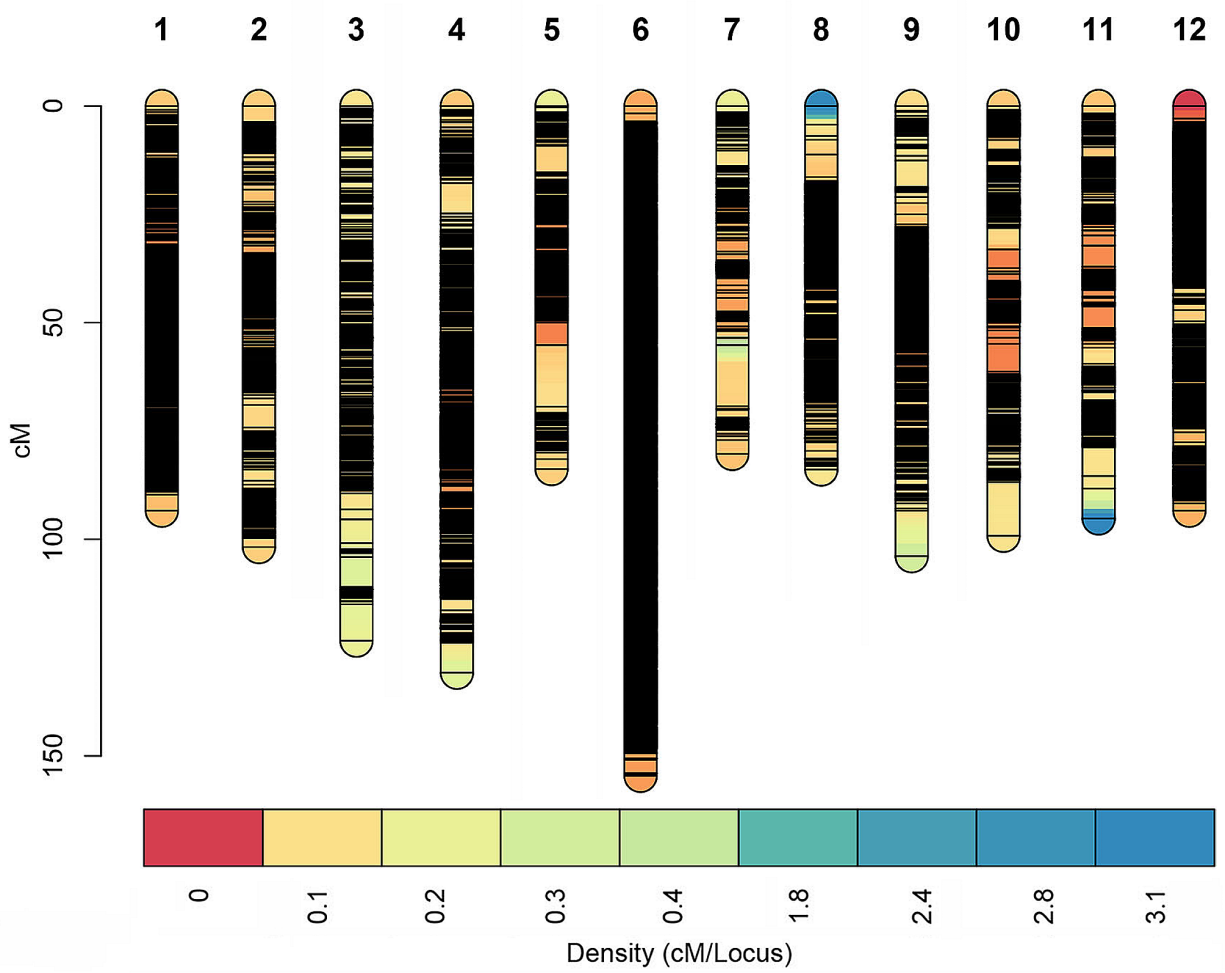
Genetic linkage map illustrating the distribution of the 249,327 markers obtained across the 12 linkage groups of goji berries used for quantitative trait locus analysis

The construction of the integrated genetic map involved the use of 249,327 SNP markers, which were used to define the genetic structure within 12 LGs of the goji berry genome. The resulting genetic map covered a total distance of 1243.74 cM, with an average distance between markers of 0.005 cM (Fig. 3). The largest LG in the integrated genetic map was LG06, which encompassed a total of 73,115 markers. This LG had a length of 154.07 cM, with an average distance between markers of approximately 0.002 cM. In contrast, the smallest LG was LG03, which contained 2109 markers that spanned 123.43 cM, resulting in an average interval of 0.059 cM. The largest gap between neighbouring markers was observed in LG05, which stretched across 14.16 cM. The ratios between successive markers in terms of genetic distance less than 5 cM varied between LGs, ranging from 97.04% for LG10 to 100% for LG03. A detailed overview (the total number of markers), the total genetic distance, and the average distance between markers are presented in Table 2.

**Table 2:** Basic characteristics of the high-density genetic map of goji berry

Linkage Group	Marker	Length (cM)	Average Spacing (cM)	Max Gap (cM)
Chr01	30,955	93.40	0.003	3.742
Chr02	9003	101.76	0.011	5.191
Chr03	2109	123.43	0.059	8.412
Chr04	16,006	130.84	0.008	6.941
Chr05	21,990	83.75	0.004	14.159
Chr06	73,115	154.61	0.002	3.165
Chr07	8737	80.27	0.009	14.16
Chr08	6386	83.95	0.013	5.19
Chr09	12,204	103.93	0.009	10.497
Chr10	21,962	99.16	0.005	12.312
Chr11	16,988	95.25	0.006	6.941
Chr12	29,872	93.40	0.003	2.877
Overall	249,327	1243.74	0.005	14.16

The quality of the constructed genetic map was evaluated using two primary methodologies, namely collinearity analysis and recombination rate distribution analysis. Heatmaps were generated to assess the quality of the genetic map by visualizing the recombination frequencies between markers within each LG using pairwise recombination rates. The heatmap accurately represented the order of the markers on the genetic map, and pairwise recombination rates between adjacent markers were found to be significantly lower (Supplementary Figure S1). To evaluate the quality of the genetic map, collinearity analysis was performed between each LG and the reference genome of *L. barbarum*. This analysis helps to assess the level of agreement and alignment between the genetic map and the reference genome. The results of the collinearity analysis are presented in Supplementary Figure S2 and Supplementary Table S4. The average Spearman coefficient, which measures the correlation between the genetic map and the reference genome, was 0.83, indicating a relatively strong degree of collinearity between the genetic map and the reference genome. Specifically, chromosomes 3, 6, and 10 exhibited higher levels of collinearity, with Spearman coefficients exceeding 0.96, suggesting a strong agreement and alignment between the markers in these LGs and their corresponding positions in the reference genome. However, chromosomes 10 and 7 displayed lower consistency, with Spearman coefficients of 0.67 and 0.69, respectively, indicating some discrepancies or deviations between the genetic map and the reference genome in these regions.

### QTL mapping analysis

**Fig. 4:**
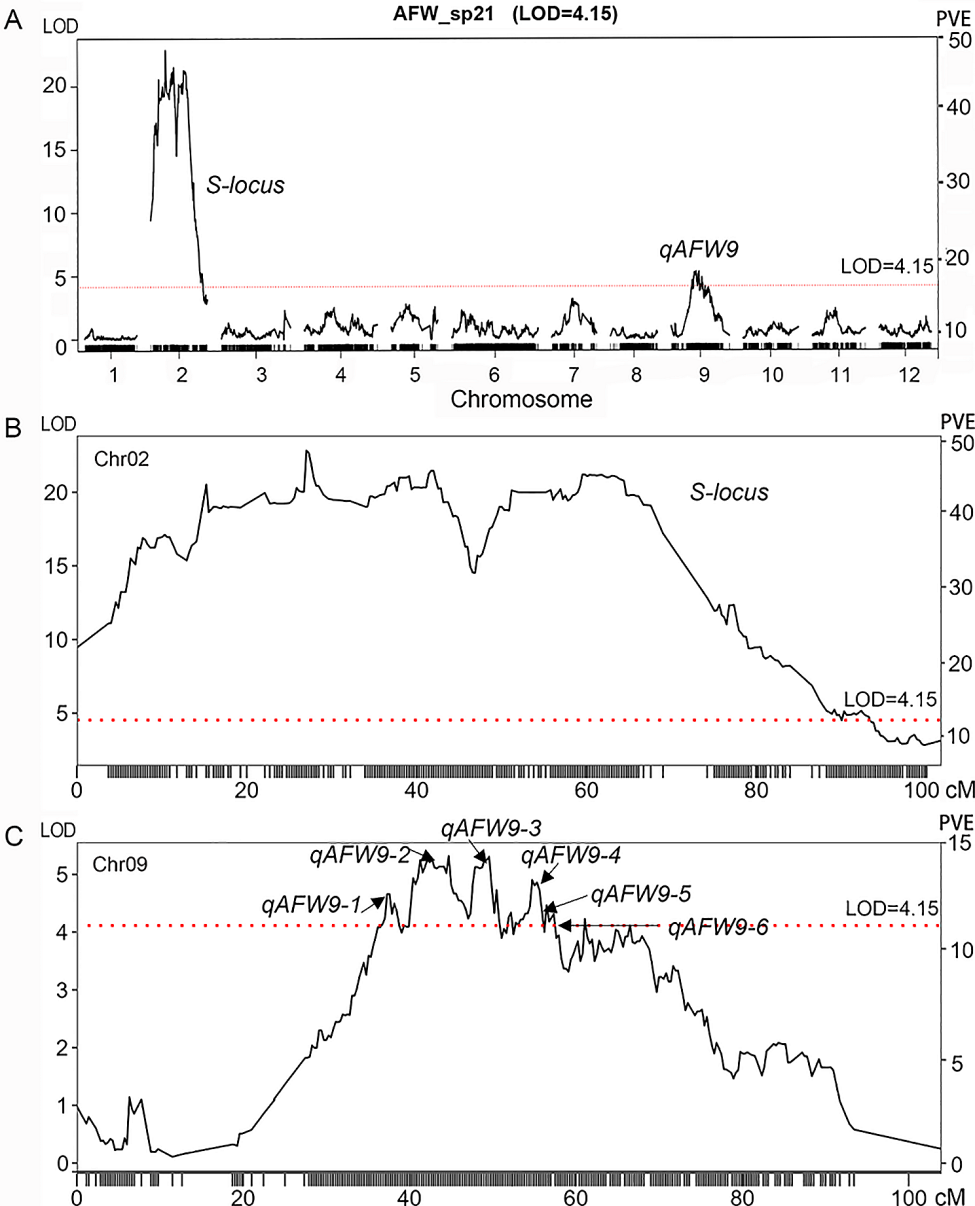
R/QTL analysis of the average fruit weight (AFW) after self-pollination in 2021. **(A)** Total QTLs. **(B)** The obtained QTLs on chromosome 2 (*S*-locus). **(C)** The obtained QTLs on chromosome 9. The x-axis indicates the genetic distance of the goji linkage groups (LGs) and the y-axis represents the likelihood ratio (LOD) score. The dashed line represents the significance threshold value of 4.15 (*P* = 0.05)

Using the high-density genetic map and the phenotypic variations among progeny, a QTL analysis was conducted using the composite interval mapping model in R/qtl. A significance threshold of *P* = 0.05 was established based on 1,000 permutations to determine the significance of each marker. A total of 43 significant QTLs were identified, with LOD values ranging from 4.22 to 50.51 (Supplementary Table S5). Collectively, these loci accounted for more than 11% of the observed phenotypic variations. Analyses of the 43 QTLs obtained from different traits across different years revealed that 37 QTLs located on chromosome 2 likely represented the same QTL. The 6 QTLs located on chromosome 9 represented another QTL, indicating that there were two distinct QTLs, one on chromosome 2 and one on chromosome 9 (Fig. 4). Notably, the specific QTL on chromosome 2 was consistently detected across different years, regardless of the relationship between self-pollination and geitonogamy (Supplementary Figure S3). The LODs of LG02 were significantly greater than those of the other chromosomes, with the strongest QTL peak (LOD score = 50.51) displaying the highest LOD score and an associated percentage of variance explained as high as 88.86%. This QTL region, known as the *S*-locus, is believed to be crucial in regulating traits associated with self-compatibility.

### Identification of S genes within the QTL localization interval based on transcriptome data

**Fig. 5:**
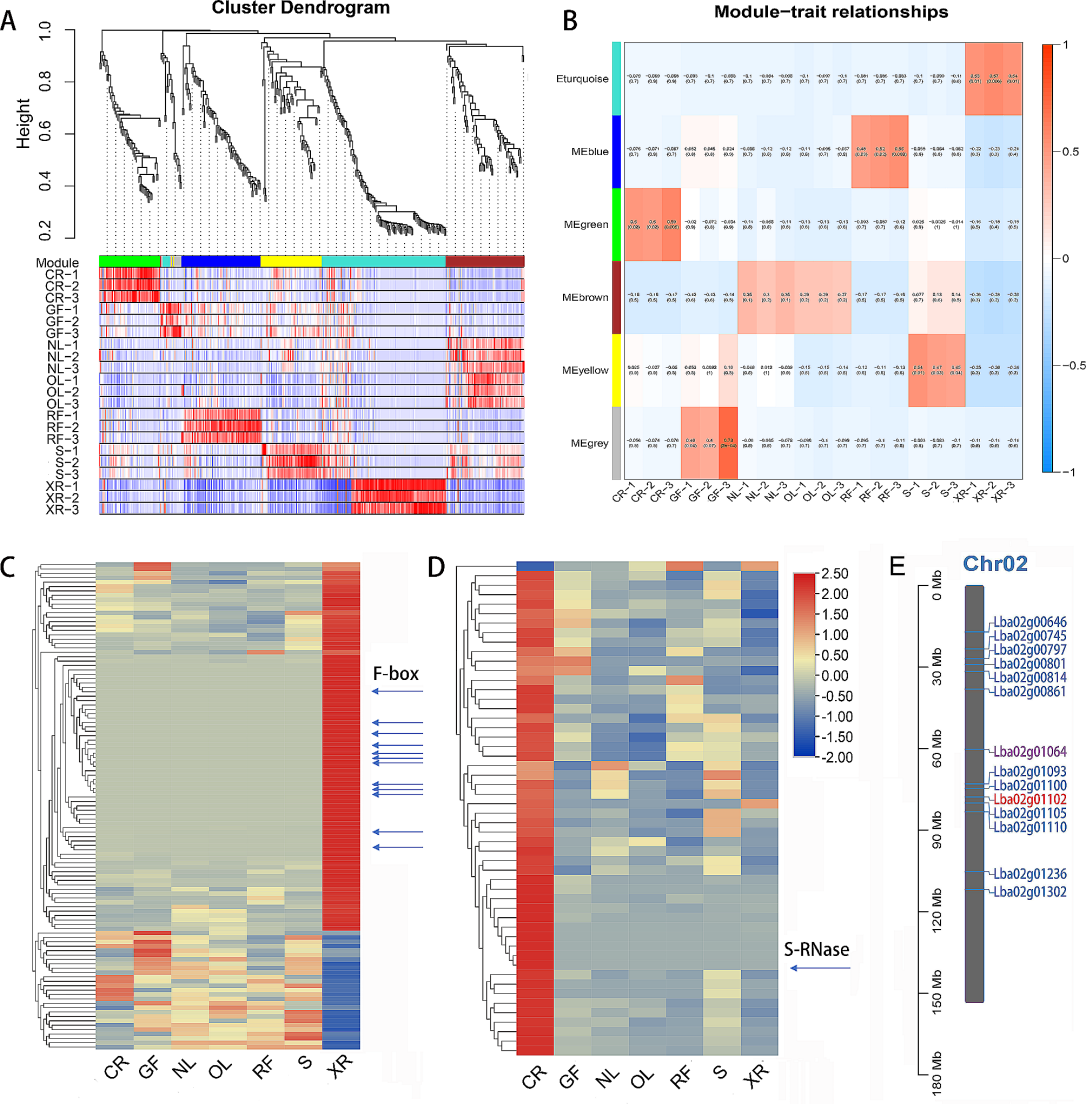
Module analysis and differential expression analysis of the annotated genes within the candidate localization interval. **(A)** Gene clustering diagram within the candidate interval. **(B)** Correlation heatmap between gene co expression modules and phenotypes. **(C)** Differential expression analysis of genes in the ME turquoise module. The arrow indicates the F-box genes. **(D)** Differential expression analysis of genes in the ME green module. The arrow indicates the *S-RNase* gene. **(E)** The relative positions of Lba02g01102 (*S-RNase*), Lba02g01064 (*AAO*), and other putative *SLF* genes on the chromosome 2

All the traits were mapped to the same interval on chromosome 2, ranging from 5.47 to 74.18 cM, and covering a total length of 68.71 cM. After annotating the candidate interval with genetic variations and Swissprot functional annotations, a total of 1180 genes (*Lba02g00358*-*Lba02g01564*) were identified and transcriptome sequencing provided expression data for these genes in different organs. Genes with zero expression were excluded, followed by a WGCNA that identified four distinct modules among the 1180 genes (Fig. 5a). The gray module (MEgray) represents a gene set that did not cluster into any other module. After the gene modules were associated with the phenotypic data, a correlation heatmap between the modules and phenotypes was generated (Fig. 5b). The turquoise module, consisting of 116 genes, showed a significant positive correlation (*r* = 0.55, *P* < 0.05) with stamen development and function, indicating a strong association between this module and stamen growth and functionality (Fig. 5c). Furthermore, the gene expression heatmap for the green module (me green) in Fig. 5d, consisting of 53 genes, exhibited a significant positive correlation (*r* = 0.53, *P* < 0.05) with pistil development and related functions. Among the 53 genes associated with pistil development, only *Lba02g01102* was expressed exclusively in pistils at a remarkably high expression level (fragments per kilobase of exon model per million mapped fragments, FPKM, value of 19,116), and functional annotation revealed it to be an *S-RNase* gene. The 116 genes associated with stamen development were mostly stamen-specific genes, with Lba02g01064 exhibiting a high expression level in stamens (FPKM value of 7726.7) and annotated as L-ascorbate oxidase (AAO). This gene is important in cell wall synthesis, plant cell death, antioxidant defense, and signal transduction [31, 32]. In addition, 12 stamen-specific genes annotated as *F-box* genes, including Lba02g00646, Lba02g00745, Lba02g00797, Lba02g00801, Lba02g00814, Lba02g00861, Lba02g01093, Lba02g01100, Lba02g01105, Lba02g01110, Lba02g01236, and Lba02g01302, may function as S-determining factors (S-locus F-box, SLF) in stamens and could be the focus of future research. Figure 5e shows the relative arrangement positions of Lba02g01102 (*S-RNase*), Lba02g01064 (*AAO*), and other putative *SLF* genes on the chromosome 2.

### Cloning of the *S-RNas*e genes in parents and analysis of *S-RNase* genotyping in the F1 population

**Fig. 6:**
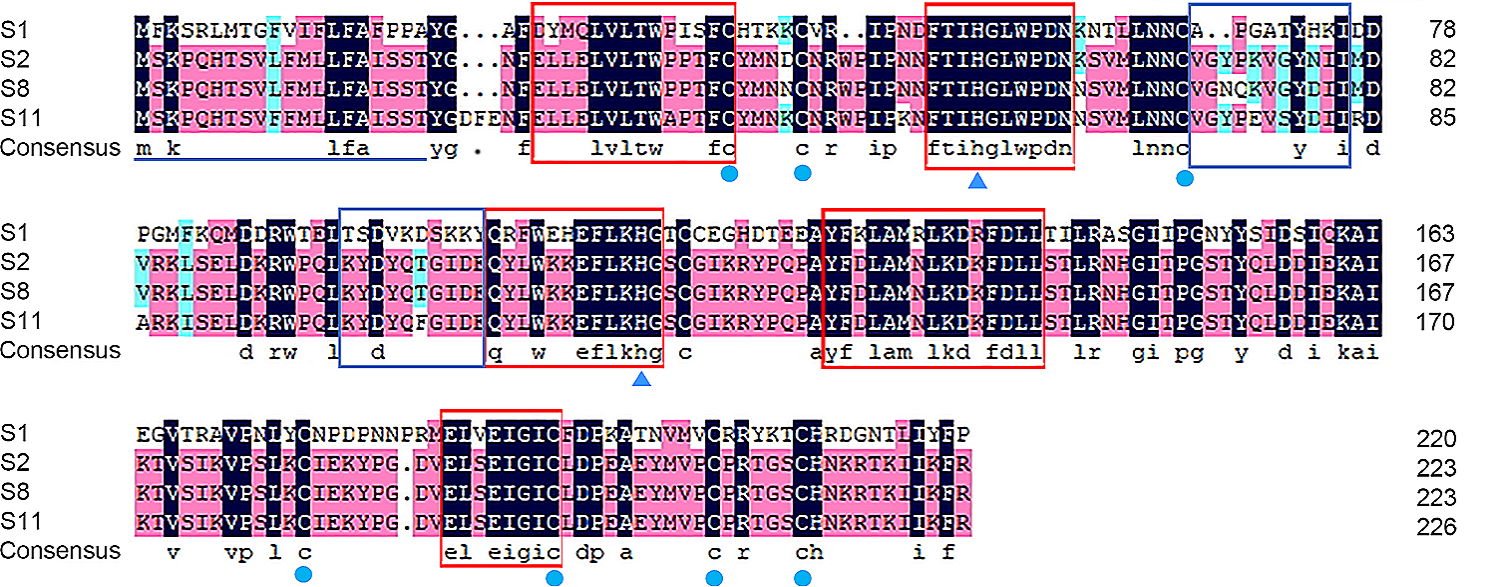
Analysis of the sequence structure of the S-RNase protein obtained from the parents. The protein sequence contains two conserved histidine residues (triangles), seven cysteine residues (solid dots), one signal peptide (underlined), five conserved regions (red boxes), and two hypervariable regions (blue boxes)

Due to the significance of the *S-RNase* gene (Lba02g01102) in regulating pistil self-incompatibility in various Solanaceae species, and its location within the S-locus interval of the QTL, we utilized homologous cloning and RACE methods to obtain *S-RNase* sequences from the parental lines ‘13–19’ and ‘new 9’. Our analysis revealed that the *S* genotypes of ‘13–19’ and ‘new 9’ were *S2S8* and *S1S11*, respectively. The coding sequences of the genes varied in length from 645 to 672 bp and the genes encoded proteins consisting of 214 to 223 amino acids. The alignment of the protein sequences revealed the presence of two conserved histidine residues and seven conserved cysteine residues. In addition, a signal peptide comprising approximately 20 amino acids was encoded at the N-terminus. Two hypervariable regions (HVa and HVb) and five conserved regions (C1-C5) in the *Lycium S-RNase* protein were consistently identified (Fig. 6). All candidate *S-RNase* genes featured a solitary intron located exclusively between the two hypervariable regions.

We extracted DNA from the offspring and used gene-specific primers to determine their genotypes. The segregation ratio of the *S-RNase* genotypes in the offspring was *S1S2*: *S1S8*: *S2S11*: *S8S11* = 65: 88: 81: 86, and the segregation ratio fit a 1:1:1:1 ratio (χ^2^ = 4.075, *P* > 0.05) (Supplementary Table S6 and Supplementary Figure S4-S8). The correlation between *S-RNase* genotypes and the SI phenotype was analyzed using Pearson correlation analysis, which indicated a significant correlation (*P* < 0.001) between *S* genotypes and the relative self-compatibility index (CCI). All offspring containing *S8-RNase* exhibited self-compatibility, while self-incompatible plants lacked *S8-RNase*.

## Discussion

### High-density genetic map of goji berries

For species with known genome sequences, many SNPs, InDels, structural variations, and other information can be found by resequencing the whole genomes of individuals and populations [33]. With the completion of whole-genome sequencing from an increasing number of crops, along with the advancement of sequencing technology and cost reduction, it is now feasible to perform genetic analysis of crop populations through resequencing [34]. Resequencing technology has been extensively utilized in the genetic analysis of various crops such as corn, rice, wheat, rapeseed, tomato, pepper, and others [35–38].

F1 populations of forest trees are commonly used for the construction of genetic linkage maps and genetic analysis due to their long growth cycles, challenging management, and high heterozygosity in genetic composition [39]. Goji, a highly polymorphic and heterozygous tree plant, had an average heterozygosity value of 0.439 [40]. Therefore, it was deemed feasible to construct a genetic map of *L. barbarum* based on the F1 population [27–30]. In this study, we resequenced the goji genome using PRJNA640228 as the reference genome to identify a substantial number of SNPs [41]. Wide-scale genotyping was performed based on an F1 population derived from two different lines of goji berries (*L. barbarum*). A total of 8,472,676 polymorphic SNP markers were obtained, which represented eight segregation types. A high-density genetic map serves as a vital bridge between traits and the genome, providing a scaffold for anchoring sequences onto chromosomes. From the markers obtained through this study, an integrated genetic map was constructed, which consisted of 249,327 markers, encompassing a genetic distance of 1243.743 cM, with an average distance between markers of 0.005 cM. However, in the integrated map, a maximum gap of 14.159 cM was specifically observed in LG05, suggesting the possibility of recombination events or the absence of developed markers in this specific region. Furthermore, compared to the previous genetic map of goji berries, the current map showed a greater total number of SNP markers and a smaller average spacing [25–28]. These results indicate that the resequencing approach utilized in the present study achieved a greater resolution than the reduced-representation sequencing method employed in the previous map. We generated a more comprehensive genetic map for *L. barbarum* than that constructed by Zhao et al. in 2021 by resequencing, with a higher number of markers and a smaller average distance between them [28]. The quality of the integrated map was evaluated using a heatmap of recombination rates, which revealed extremely low recombination rates between adjacent markers. The recombination rate between paired markers gradually increased with increasing in transmission distance, indicating that the SNP markers in the linkage group were well ordered and that the integrated map constructed in this study had high accuracy. This genetic map could result in more recombination events and increase the accuracy of QTL positioning.

### QTL analysis for SI related traits

To prevent inbreeding and increase genetic diversity, certain plant species have developed a parallel mechanism known as the SI response, which serves to prevent self-pollination or the deposition of genetically similar pollen on the surface of the stigma [4]. The SI system depends on a network of genes essential for recognizing and transmitting signals and either rejecting or accepting self-pollen. The specific interactions between these loci and their roles in the downstream signaling cascade of SI are under investigation; however, QTLs for self-(in)compatibility traits have been successfully mapped or finely mapped in many crop species.

In addition to a dependent genetic map, a precise evaluation of the trait is crucial for QTL mapping. In ryegrass (*Lolium perenne* L.), several QTLs and candidate genes linked to self-compatibility have been identified using data from in vitro pollination assays [18–24, 42]. To assess the SI trait in sunflower, manual selfing was performed, and the number of seeds produced per secondary capitula was counted [19]. In the study of sporophytic SI *Silphium integrifolium* (Asteraceae), a set of seeds, calculated as the ratio of fertilized to total ovules, above 20% was considered to indicate compatibility [43]. In this study, four indicators related to the SI were used for each evaluation method, namely, the FR, AFW, CI, and CCI. These four SI indices exhibited significant correlations. The Pearson correlation coefficient between the CCI and CI was 0.98, that between the FR and CCI was 0.78, and that between the AFW and CCI was 0.64. To comprehensively assess the SI trait of goji, in this study two methods were used: Self-pollination and geitonogamy. Although most F1 plants exhibited consistent performance based on the four indicators assessed, some F1 plants displayed inconsistencies between the two pollination methods. After pollination, pollen adheres to the surface of the stigma and undergoes germination in the pollen tubes following hydration of the stigma, which is a crucial step in the mutual recognition process between pollen and stigma [44]. For example, both self-pollinated and geitonogamous pollen adhere to the stigma in alfalfa [45]. However, the pollen germination rate is lower under self-pollination than under geitonogamy. Moreover, the time taken for the pollen tube to enter the embryo sac is longer under self-pollination than under geitonogamy in alfalfa [45]. The factors that influence plant self-compatibility are highly complex and can include pollen germination, growth and development of the pollen tube, successful fertilization, and ovule maturity. The growth status of the pollen tubes within the style and their ability to enter the ovary to complete fertilization both via self-pollination and geitonogamy remain unclear. Therefore, future studies should establish a theoretical foundation to improve the rate of artificial pollination and fertilization.

The most found goji berry varieties, including the wild type, are self-incompatible. However, there are a few self-compatible varieties. As a result, the reasons behind the transition from self-incompatible to self-compatible goji berries, as well as the genetic mechanisms responsible for controlling self-incompatibility in goji berries, remain unknown. To bridge this knowledge gap, our objective was to map the genomic position of the self-recognition locus (S-locus) and SI related loci in this species. The present QTL mapping analysis successfully identified the S locations in LG2 of *L. barbarum*. Perhaps due to the strong S-locus effect, we have not discovered any other effective SI-related sites beyond the S-locus.

### Analysis of candidate genes

Within the interval region of the S-locus, a potential candidate gene, Lba02g01102, was found to be the *S-RNase* gene. S-RNase-based gametophytic SI is observed in various plant families, including Rosaceae, Solanaceae, Scrophulariaceae, and Rubiaceae. Interestingly, despite the use of similar genes by different taxa to determine the specificity of pollen rejection, notable differences exist in the specific involved mechanisms. In various plant families, the *S* locus commonly comprises a minimum of two linked genes and, in many cases, even more. Among these genes, one of the key components is *S-RNase*, which is a glycoprotein expressed in the pistil that possesses ribonuclease activity. Acting as highly selective cytotoxins, S-RNases are crucial in the recognition process. When the single S-haplotype of pollen matches one of the two S-haplotypes present in the diploid pistil, S-RNases lead to pollen rejection [46, 47]. An abnormality in the pistil S factor can potentially lead to a transition from SI to self-compatibility in plants [48, 49]. For example, in citrus, a gene mutation caused by a single base deletion in the *Sm-RNase* gene results in a frameshift mutation, leading to self-compatibility [50].

We are the first to clone the full-length sequences of four *S-RNase* genes from parents and use gene-specific primers to identify the S genotype of the offspring. The results indicated that the *Lycium* S-RNase protein is similar to that of other Solanaceae plants, with two hypervariable regions (HVa and HVb) and five conserved regions (C1-C5) identified [51]. Two conserved histidine residues and seven conserved cysteine residues were found, and a signal peptide consisting of approximately 20 amino acids is encoded at the N-terminus. No changes were observed in the conserved amino acid positions. Analysis of the association between offspring genotype and phenotype also revealed a significant correlation between *S-RNase* gene type and SI. All these findings suggest that S-RNase may be the S pistil factor responsible for controlling SI.

Ascorbate oxidase, a protein widely found in plants and fungi, has been suggested function in regulating plant stress responses and developmental processes [31, 32]. However, its precise biological function is largely unknown. In this study, we made a fascinating discovery regarding the Lba02g01064 gene, which is annotated as an L-ascorbate oxidase. This gene displayed a significantly high expression level in stamens, with an FPKM value of 7726.7. In contrast, its expression in other organs and tissues remained relatively low, with an FPKM value of approximately 10. These findings suggest that the Lba02g01064 gene may be vital in pollen growth and development, as well as in the recognition process between pollen and the stigma.

Another relevant candidate gene identified within the interval was Lba02g00861, which was annotated as an F-box protein. F-box proteins are important components of the plant ubiquitin proteasome system and typically form complexes of Skp1-CUL1-F-box (SCF) with Skp proteins, CUL proteins, and Rbx1 proteins [52, 53]. These complexes can recognize substrate proteins and target them for ubiquitination and degradation, thus influencing various important physiological processes in plants, including embryogenesis, floral development, plant growth and development, responses to biotic and abiotic stresses, hormonal responses, and senescence [54]. The F-box protein is also crucial in controlling the recognition of SI between the pollen and pistil. In S-RNase-based gametophytic SI systems, represented by families such as Rosaceae, Solanaceae, Scrophulariaceae, and Rubiaceae, the *SLF* genes are recognized as male determinants. The SCF complex can specifically recognize and bind nonself S-RNase through SLF (S-locus F-box) proteins, resulting in ubiquitination of S-RNase and subsequent degradation by the 26 S proteasome. However, self S-RNase is not recognized and therefore remains intact, acting as a cytotoxic agent to degrade pollen tube RNA and triggering a self-incompatibility response [55]. Used large-scale genomic sequencing, researchers have cloned *SLF* genes near the *S-RNas*e gene in various plants such as Solanaceae, Petunia, and Rosaceae [56–58]. The Solanaceae plants and Rosaceae apple subfamily plants have up to 16–20 *SLF* genes near their *S* locus, all of which serve as pollen S determinants. Through their collaborative action, these SLF proteins can recognize different nonself S-RNases, thus neutralizing the toxicity of foreign S-RNases [50, 59–62]. In fact, in the QTL loci, 11 additional F-box genes near *S-RNase* were found, in addition to *Lba02g00861*. These F-box genes can potentially act as male determinants and work together. In addition, a non-S factor F-box gene called *Sli* has been discovered in potato (*Solanum tuberosum* L.). The *Sli* gene functions by inhibiting the activity of *S-RNase*, resulting in the transformation of the originally self-incompatible potato into self-compatible potato [14, 15]. These studies and findings also confirm the crucial role of F-box genes in self-incompatibility. Future studies are warranted to clone these F-box genes and investigate their functions. The other QTL, located within the range of 37.10 cM to 57.51 cM on chromosome 9, was annotated with 949 genes. This QTL was specifically associated with the average fruit weight trait in the 2021 self-pollination combination and may be unstable.

In future work, our goal will be to expand the population for fine mapping while cloning and functionally validating the candidate genes S-RNase and SLF in goji berries. For gene function verification, we will investigate the linkage between specific *SLFs* and *S-RNase* genes in F1 populations. Additionally, we plan to employ CRISPR technology to create mutant plants with alterations in S-RNase or SLF expression to examine potential changes in the SI. Through these efforts, we aim to gain a deeper understanding of whether S-RNase and SLF serve as determinants of male and female determinants in goji berries.

## Conclusions

In this study, a high-resolution genetic map was generated using genome resequencing to detect de novo SNPs in 229 F1 individuals derived from the self-compatible *L. barbarum* variety ‘13–19’ and the self-incompatible variety ‘new 9’. The genetic map consists of 249,327 SNPs distributed across 12 LGs, spanning 1243.74 cM with an average interval of 0.002 cM, with the *S* locus assigned to LG2. A total of 1180 genes were annotated within the localization interval, including Lba02g01102 (annotated as an *S-RNase* gene), which showed pistil-specific expression and may serve as the S-determining factor for the pistil. Additionally, 12 F-box genes with stamen-specific expression may function as S-determining factors for the stamen. Furthermore, Lba02g01064 (annotated as L-ascorbate oxidase) with stamen-specific expression may also be related to the self-incompatibility control in goji. Each parent contributed two different *S-RNase* genes, and the analysis of the *S-RNase* genotype in the F1 population revealed a significant correlation between the *S-RNase* genotype and self-incompatibility. These results provide valuable information on the genetic mechanisms underlying self-compatibility in goji berries and support the implementation of marker-assisted selection techniques in goji breeding programs. Furthermore, these findings pave the way for targeted breeding strategies to improve goji berry varieties.

## Materials and methods

### Plant materials

A linkage map was created using an F1 population consisting of 229 individuals of *L. barbarum*. These individuals were obtained by crossing two selected genotypes, ‘13–19’ and ‘new 9’, which were selected based on their desirable agronomic traits and contrasting SI characteristics. The ‘new 9’ was originated from the F1 progeny of (*L. barbarum* ‘0701’ × *L. barbarum* ‘Ningqi5’) × (*L. barbarum* ‘Ningqi5’ × *L. barbarum* ‘Ningqi8’), and ‘13–19’ originated from *L. barbarum* ‘Ningqi1’ × *L. barbarum* ‘Ningqi9’. A cross between ‘13–19’, a self-compatible genotype, and ‘new 9’, a self-incompatible genotype, was carried out at the Goji Institute in June 2016. Approximately 500 F1 individuals were obtained from this cross, 229 of which were randomly chosen to form the mapping population. The seeds were planted in the greenhouse in spring 2017 and subsequently transplanted to a field at the experimental base Luhuatai, Institute of Goji, Ningxia Academy of Agricultural and Forestry Sciences, Yinchuan City, Ningxia Province, China (38°38′N, 106°9′E) in May.

### Phenotypic traits estimation

The pollination experiment involved both self-pollination and geitonogamy using pollen from the same plant. Geitonogamy, performed one day before anthesis, involves collecting unopened buds as pollen donors. On the following day, flowers that had open blooms with unopened anthers were revisited and their anthers were removed to prevent self-pollination. These flowers were then pollinated with pollen collected from the same plant. Pollen was obtained from more than 10 donors and each flower in the treatment group was pollinated using a minimum of three flowers from this donor pool. Following pollination, the flowers were again covered with paper bags to prevent external pollen and insects from affecting the experiment. For self-pollination, the unopened buds of each plant were covered with paper bags. A total of 25 self-pollinations and 25 geitonogamy events using pollen from the same plant were performed on each F1 individual every May.

In July, the ripe fruits within the paper bags were collected and the number of paper bags harvested and the number of fruits were recorded. Fruits were weighed and scanned, and their seeds were extracted and counted.

The fruit setting rate (FR) was calculated as the number of fruits harvested per plant divided by the number of paper bags recycled. The average fruit weight (AFW) was determined by dividing the weight of the fruit harvested per plant by the total number of paper bags recycled. The self-compatibility index (CI) was obtained by calculating the total number of seeds per plant divided by the number of recycled paper bags. The number of ovules in each ovary of each F1 plant varied. Therefore, the average number of ovules in each ovary of each F1 plant was determined in August 2021. This was achieved by taking five unopened flowers from each plant, peeling the ovaries under an anatomical microscope, and counting the number of ovules. The average number of ovules from the five flowers was then calculated. The compared self-compatibility index (CCI) was calculated based on the number of ovules, using the formula CCI = (CI/ovules per fruit) × 100%. These traits were evaluated over two individual years (2021–2022), as well as an additional year represented by the means of 2021 and 2022 (2021/22).

SPSS 18.0 software (SPSS, Chicago, IL, USA) was used to perform a correlation analysis of the phenotype values.

### DNA extraction, genome resequencing, and SNP development

Genomic DNA was extracted from the young leaves of the parents and the progeny using the Tiangen Biotech Genomic DNA Extraction Kit (Tiangen Biotech, Beijing, China). DNA quality was assessed on agarose gels, and the DNA concentration was measured using a Qubit 2.0 system (Multiskan FC, Thermo Scientific™, Waltham, MA, USA). A resequencing library for the qualified DNA was constructed using the GenoBaits DNA seq Library Prep Kit (Molbreeding, Shijiazhuang, China) following the manufacturer’s instructions. Following additional quality control and purification processes, the libraries were sequenced using a 150-base paired-end approach on an MGI-2000/MGI-T7 platform (Illumina, San Diego, CA, USA) at BGI Genomics Co., Ltd. (Shenzhen, China).

All the raw sequences were processed using fastp software (version 0.20.0) to obtain clean reads [63]. Clean reads were aligned to the reference genome of *L. barbarum* (PRJNA640228, https://www.ncbi.nlm.nih.gov/bioproject/640228) using BWA v0.7.15 software [64]. SNP and insertion-deletion (InDel) markers were filtered using the SelectVariants and VariantFiltration tools of GATK 3.7 [65]. ANNOVAR software was used for SNP and InDel annotations [66].

### Genetic linkage map construction and QTL mapping

The genetic map was constructed using Lep-MAP3 software (https://sourcefrorge.net/projects/lep-map3/), which is based on the maximum likelihood method, as described by Ratas (2017) [67]. To ensure the precision of the genetic map, SNPs that deviated from the expected Mendelian segregation with a significance level of *P* < 0.001 were excluded from the analysis. The total length of the genetic maps was estimated using the Kosambi mapping function, first proposed by Kosambi in 1943 [68]. This function is commonly used to convert recombination frequencies to map distances in centi-Morgans (cM). The Order-Marker2 module was used to check for contiguous sequence continuity in the generated maps. Genetic maps were drawn using MadMapper (http://cgpdb.edu/Xlinkage/MadMapper).

QTL analysis was performed using the R/qtl software package (http://www.rqtl.org/) with the composite interval mapping method, following the approach described by Arends et al. (2010) [69]. The significance of each QTL interval was assessed using the likelihood ratio (LOD) statistic. To determine the LOD threshold for identifying QTLs, 1000 permutations were performed at a significance threshold of 0.05 for each environment. Candidate genes were classified using annotations from databases such as the Gene Ontology, Kyoto Encyclopedia of Genes and Genomes, and Nr databases, as outlined by Ogata et al. (1999) [70] and Ashburner et al. (2000) [71].

### Gene Co expression module identification and differential gene expression analysis

The localization interval was annotated using the SwissProt database to obtain annotated genes within this interval. Transcriptome data of *L. barbarum* from various organs, including pistils, stamens, stem tips, leaves, green fruits, and red fruits were utilized to obtain the expression levels (FPKM values) of different genes within the localization interval. Weighted gene co expression network analysis (WGCNA) was conducted to construct a co expression network to identify co expressed gene modules and explore the correlation between gene modules and different organs [72]. The modules related to the stamen and pistil were identified, and differential gene expression analysis was carried out within these modules.

### Cloning of the *S-RNase* gene and analysis of the genotype of the *S-RNase* gene in the generation of F1

The full-length gDNA sequence of *S2-RNase* from the *Lycium* genome sequence (PRJNA640228) was obtained via sequence alignment. Primers (*S2-RNase*-F: 5’-ATG TCT AAA CCA CAG CAC ACA TCA G-3’; *S2-RNase*-R: 5’-TCA TCG GAA CTT AAT TAT CTT AGT T-3’) were designed based on the genome sequence, and the complete sequence of *S2-RNase* was successfully cloned from ‘13–19’. The same set of primers was also used to clone the sequences of *S8-RNase* from ‘13–19’ and *S11-RNase* from ‘new 9’. The *S1-RNase* gene was cloned from the parent plant ‘new 9’ using homologous cloning and the SMRTer™ RACE cDNA Application Kit (Clontech, Mountain View, CA, USA). The full cDNA sequence was amplified with PrimeSTAR® Max DNA Polymerase (Takara, Dalian, China) and cloned and inserted into the pGEM-T Vector System (Promega, Madison, WI, USA).

For generation F1, all individual plants were selected, and their young leaves were collected for DNA extraction using a DNA extraction kit (Tiangen Biotech, Beijing, China). The *S-RNase* genotype of the F1 offspring was determined by PCR amplification using taq polymerase with gene-specific primers (S1-F: 5’-ATG TTT AAA TCA CGA CTC ATG ACT G-3’, S1-R: 5’-TCA CCC TGG AAA ATA AAT TAA AGT G-3’; S2-F: 5’-GCT ACC CAA AAG TGG GTT ATA A-3’, S2-R: 5’-GAG TTT GCC ATT AAT TAT GCT TAG C-3’; S8-F: 5’-GTG GGC AAC CAA AAA GTG GGT TAT G-3’, S8-R: 5’-GCC TTT TCG ATA TCA TGA AGC A-3’; S11-F: 5’-GCT ACC CAG AAG TGA GTT ATG A-3’, S11-R: 5’-CAG CTC ACT TAT TTT TCT GGC ATC C-3’) (Supplementary Figure S4-S8). PCRs were performed in a reaction mixture containing approximately 10 ng of template (genomic DNA), 1×PCR buffer, 0.25 mmol/L deoxynucleotide triphosphates, 0.25 mmol/L forward and reverse primers, and 0.5 U of Taq DNA polymerase (Qiagen, Valencia, CA, USA) in a final volume of 20 µL. PCR amplification was performed with initial denaturation at 94 °C for 4 min followed by 30 cycles of denaturing at 94 °C for 30 s, annealing at 57 °C for 30 s, extension at 72 °C for 60 s, and a final extension at 72 °C for 10 min [73].

### Electronic supplementary material

Below is the link to the electronic supplementary material.

**Supplementary Material 1: Table S1.** A combined analysis of variance (ANOVA) performed on the data collected from 229 offspring of the F1generation, involving eight phenotypic traits over two consecutive years (2021?2022)

**Supplementary Material 2: Table S2.** Statistics for the sequencing data and alignment with the reference genome

**Supplementary Material 3: Table S3.** The information on the indels presented in the CDS

**Supplementary Material 4: Figure S1.** Heatmaps showing the recombination frequencies among markers located on each linkage group to evaluate the quality of the genetic map using the pairwise recombination rate. The vertical and horizontal coordinates represented the markers located within the LG; a blue square indicated a high rate of recombination, while red squares indicated a low rate of recombination

**Supplementary Material 5: Figure S2.** Collinearity analysis of all goji linkage groups (LGs) with the goji genome sequence. The x-axis indicates the genetic distance of goji LGs; markers in these LGs are plotted as dots in the figure

**Supplementary Material 6: Table S4.** Spearman correlation coefficients between genetic and physical positions

**Supplementary Material 7: Table S5.** Quantitative trait loci for self-incompatibility-related traits detected by composite interval mapping

**Supplementary Material 8: Figure S3.** QTL analysis for all investigated self-incompatibility related traits. FR, fruitful rate; AFW, average fruit weight; CI, self-compatibility index; CCI, compared compatibility index; _sp, collected after self-pollination; _ge, collected after geitonogamy

**Supplementary Material 9: Table S6.** The S genotype of F1 population. Identified using gene-specific primers with the PCR method. Use “☑” to indicate the presence of a band corresponding to the genotype. * Means the trees randomly selected to sequencing for constructing genetic map

**Supplementary Material 10: Figure S4-S8.** PCR Amplification electrophoresis of S genotype in parents and F1 population

## Data Availability

The datasets that support the findings presented in this paper are included within the article (and its supplementary materials). The raw reads for the genome resequencing of 229 F1 progenies and their parents can be found in the NCBI SRA database under the accession number PRJNA1020911 (https://www.ncbi.nlm.nih.gov/bioproject/PRJNA1020911). Raw data for different organs of L. barbarum sequenced libraries are available in the Genome Sequence Archive of National Genomics Data Center under the accession number PRJCA025572 (https://ngdc.cncb.ac.cn/bioproject/browse/PRJCA025572).

## Abbreviations

AFLP: Amplified fragment length polymorphism
AFW: Average fruit weight
CCI: Compared self-compatibility index
CI: Compatibility index
CDS: Coding sequence
FPKM: Fragments per kilobase of exon model per million mapped fragments
FR: Fruit setting rate
LG: Linkage group
LOD: Likelihood-ratio statistic
QTL: Quantitative trait locus
SD: Standard deviation
SI: Self-incompatibility
SLAF: Specific locus amplified fragment
SLF: S-locus F-box
SNP: Single-nucleotide polymorphisms
and SSR: Simple sequence repeat
