# Supplementary figures and images for: Mapping quantitative trait loci associated with self-(in)compatibility in goji berries (Lycium barbarum)
Source: BMC Plant Biol. 2024 May 23;24:441. doi: 10.1186/s12870-024-05092-7 (PMC11112781; doi:10.1186/s12870-024-05092-7)

**LG1**

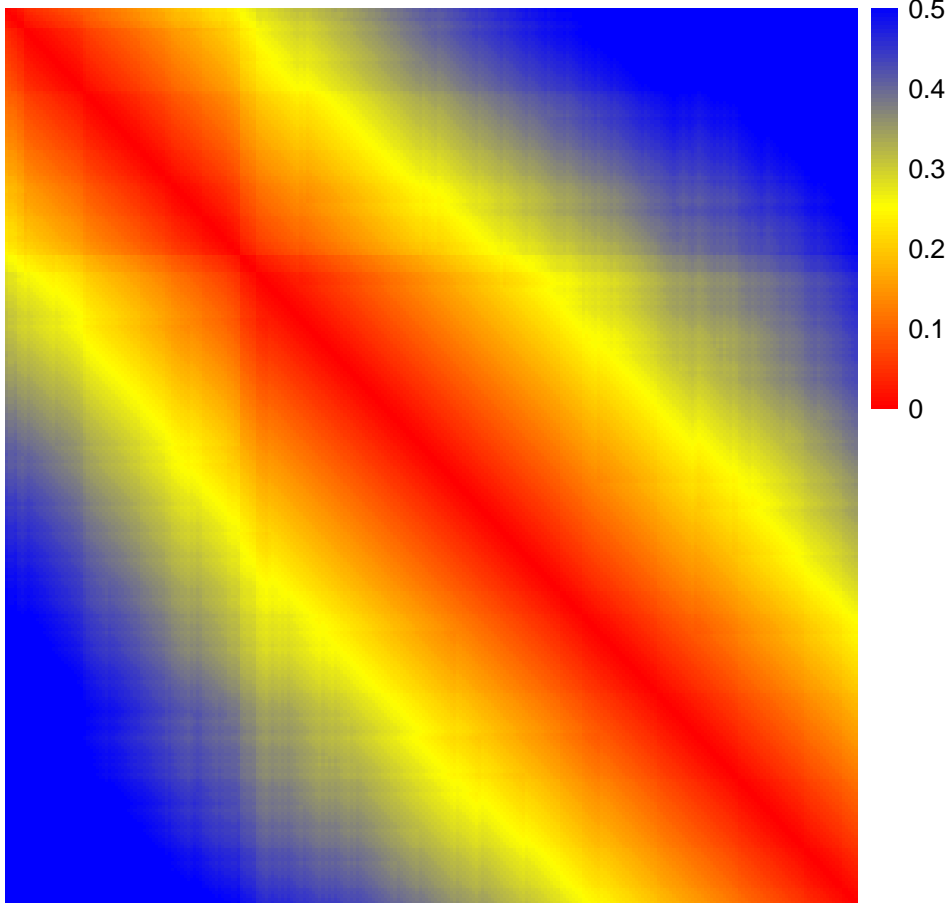

**LG2**

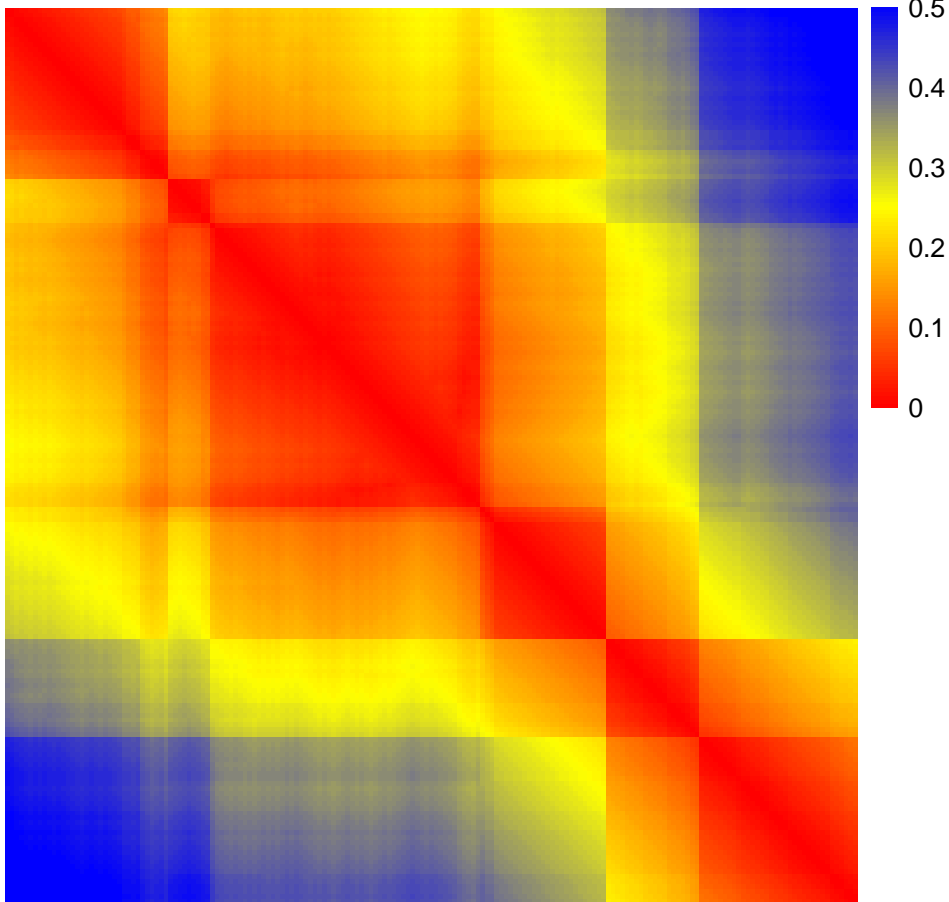

**LG3**

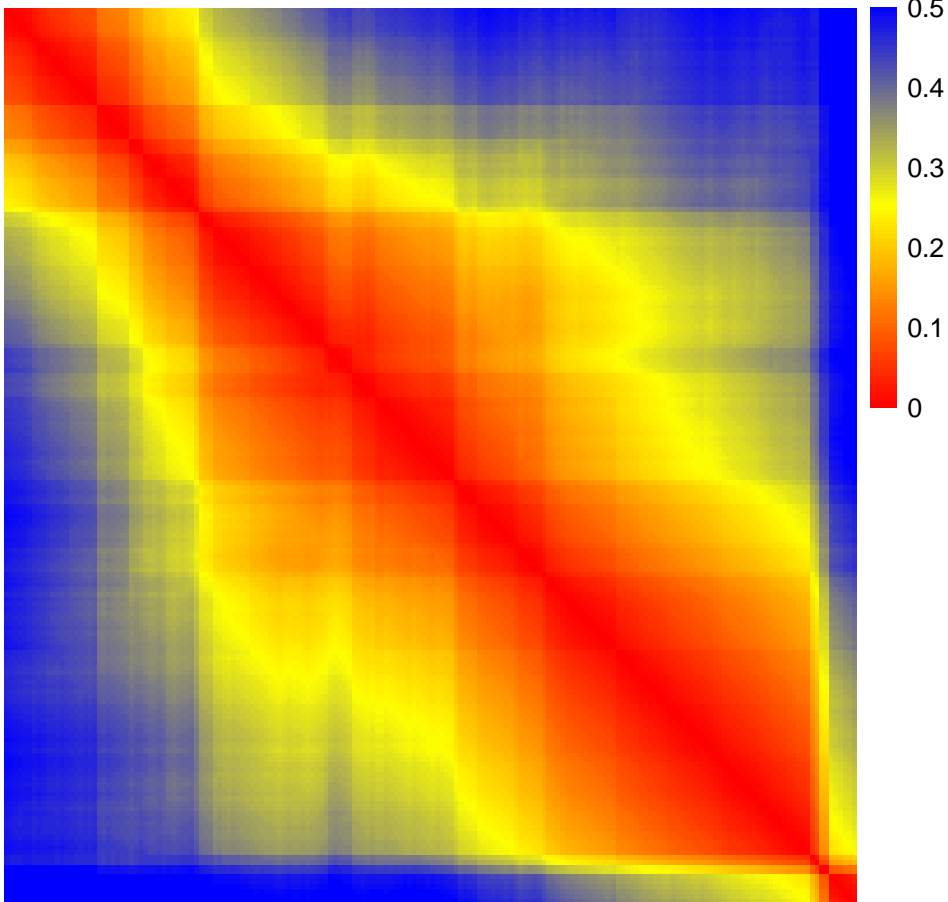

**LG4**

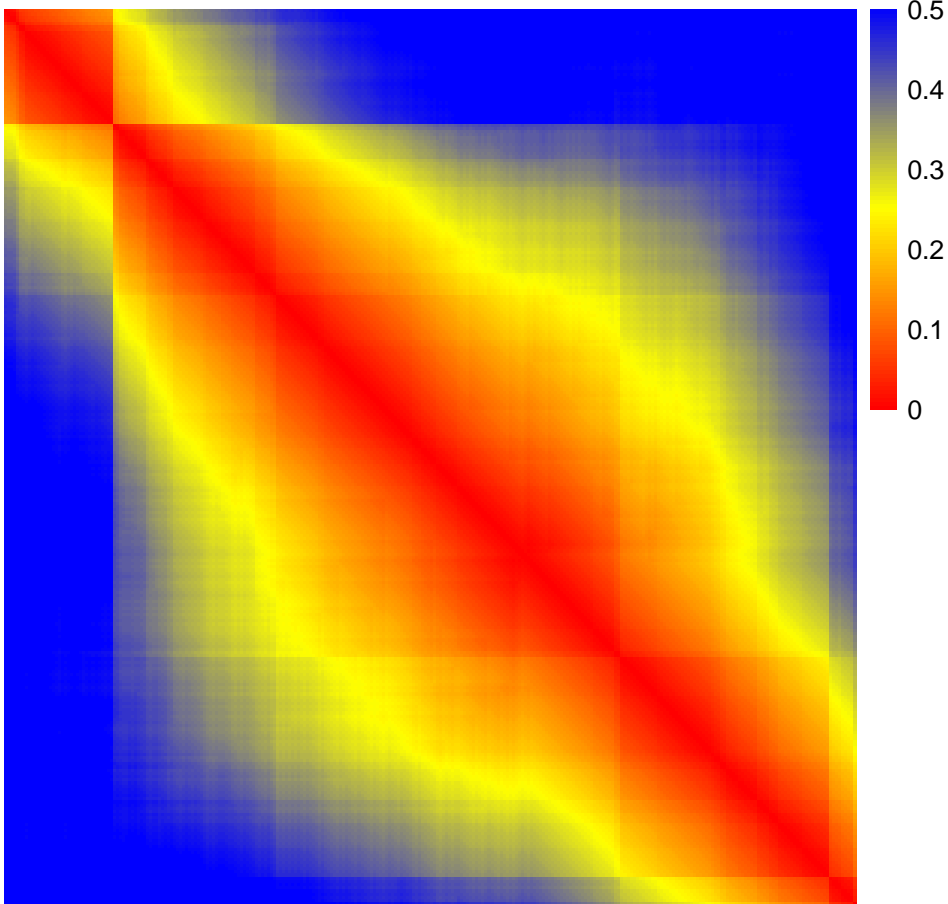

**LG5**

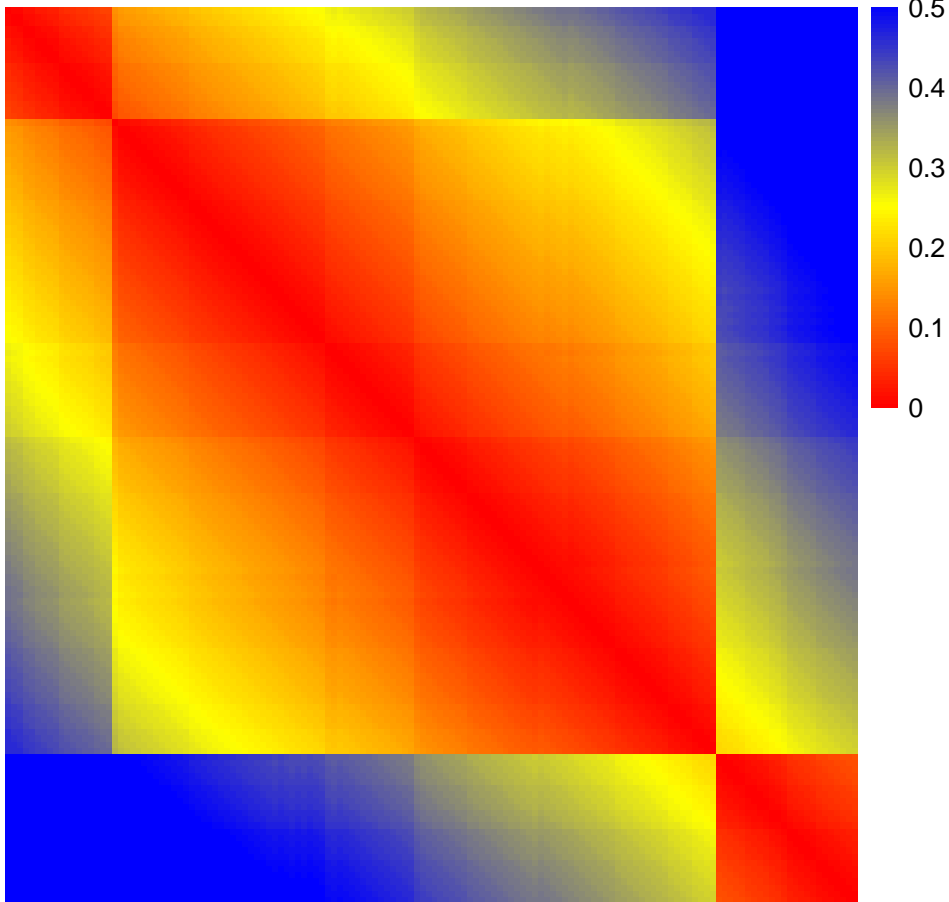

**LG6**

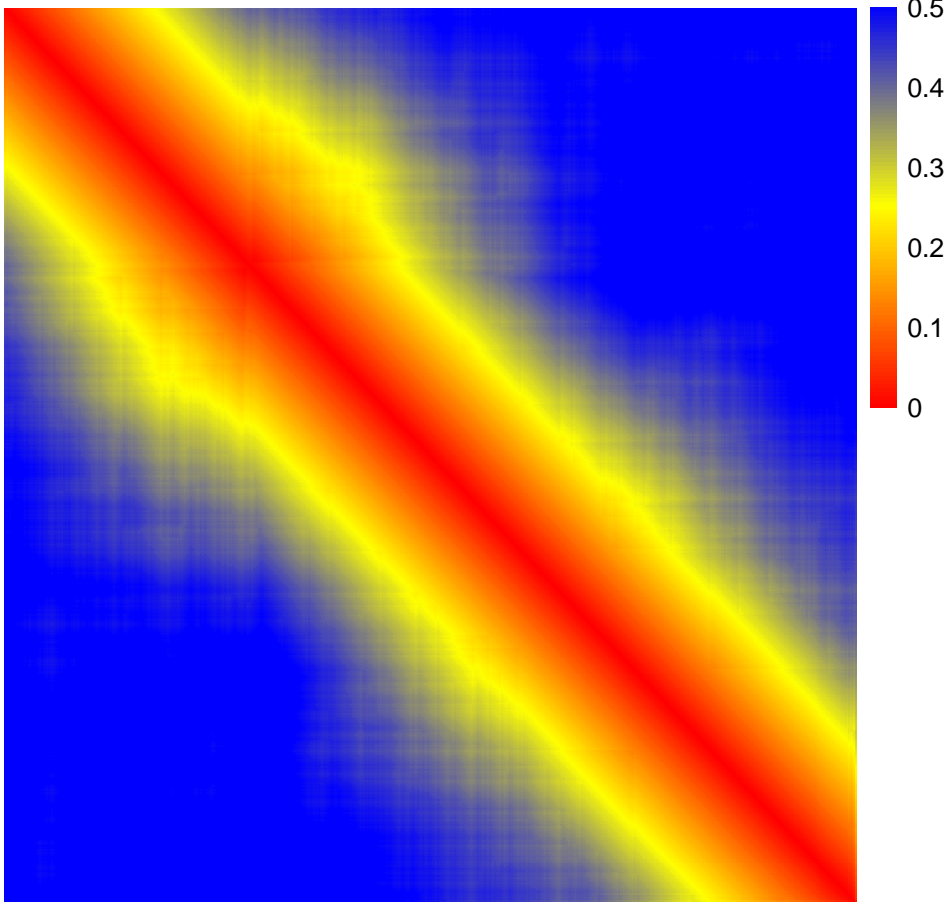

**LG7**

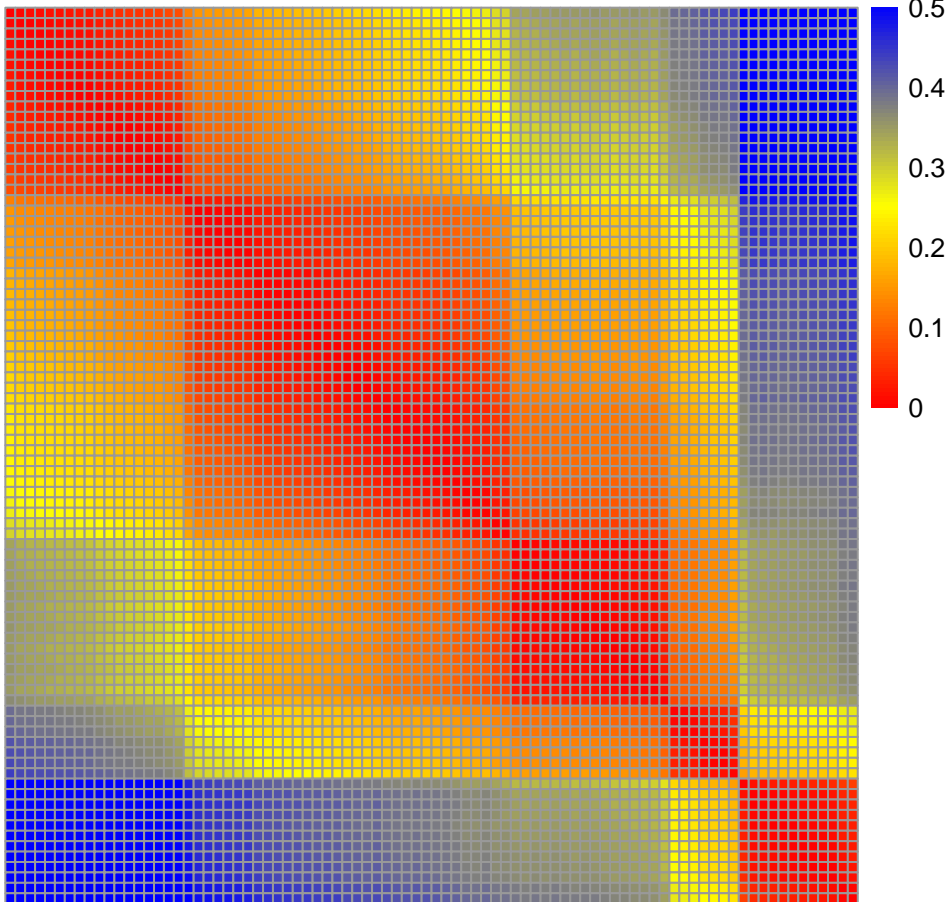

**LG8**

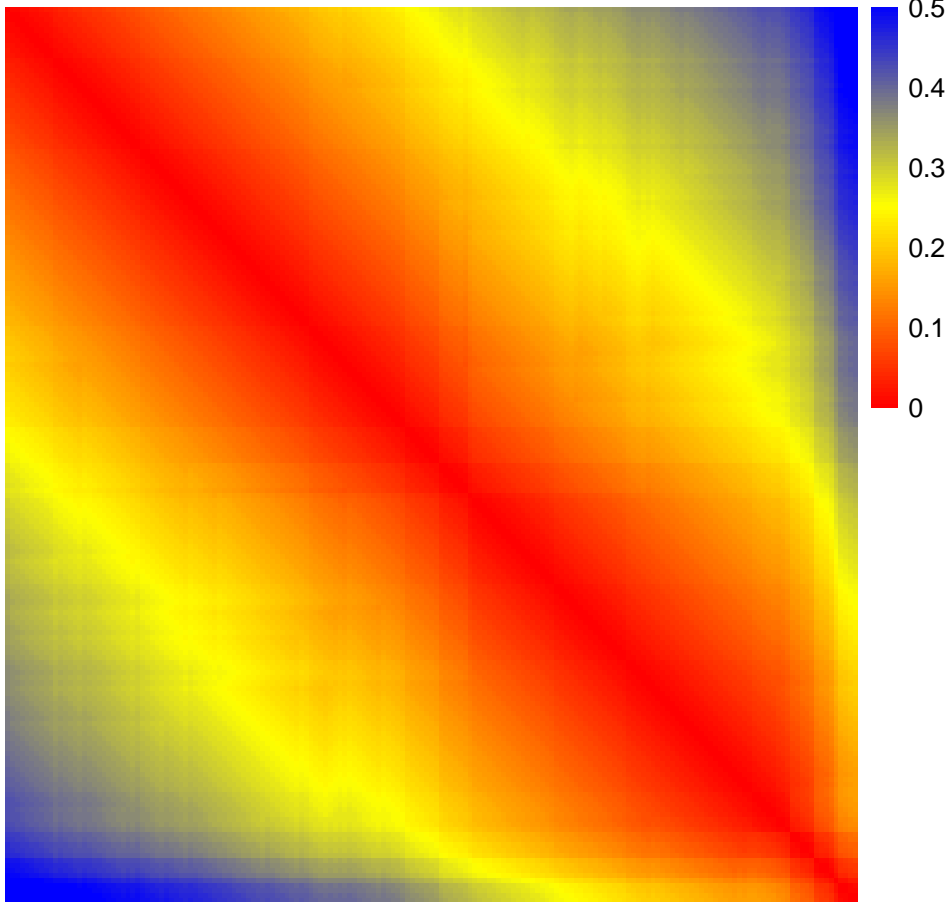

**LG9**

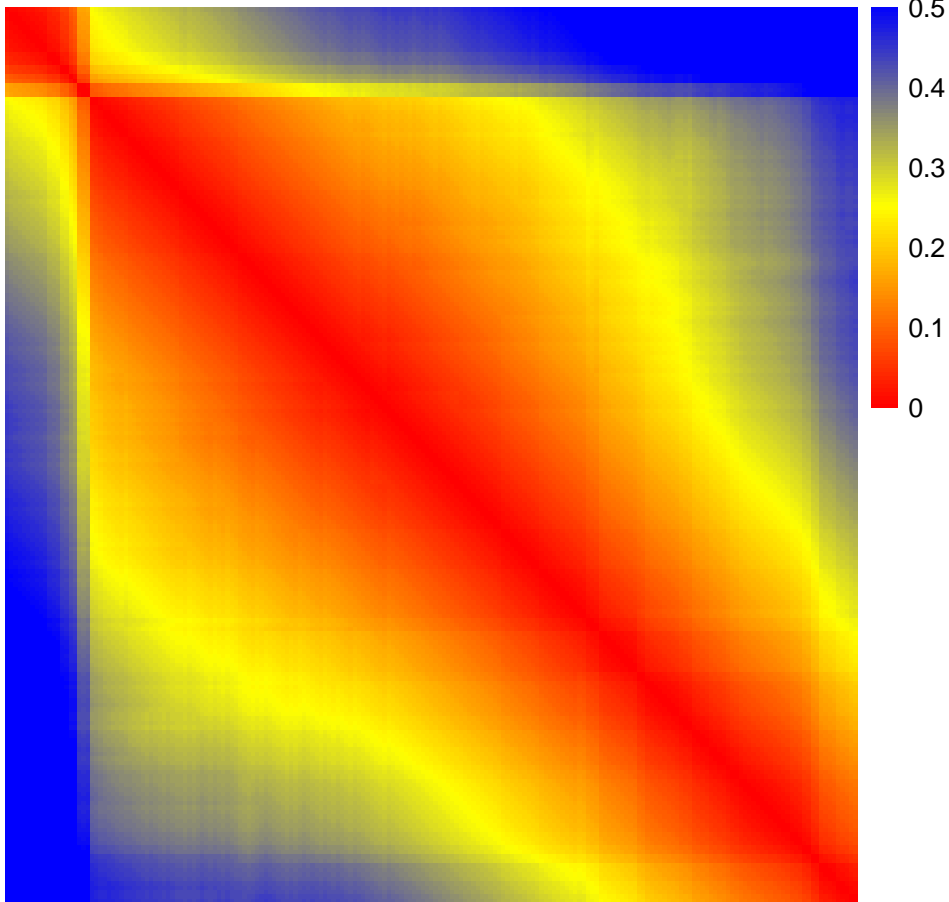

**LG10**

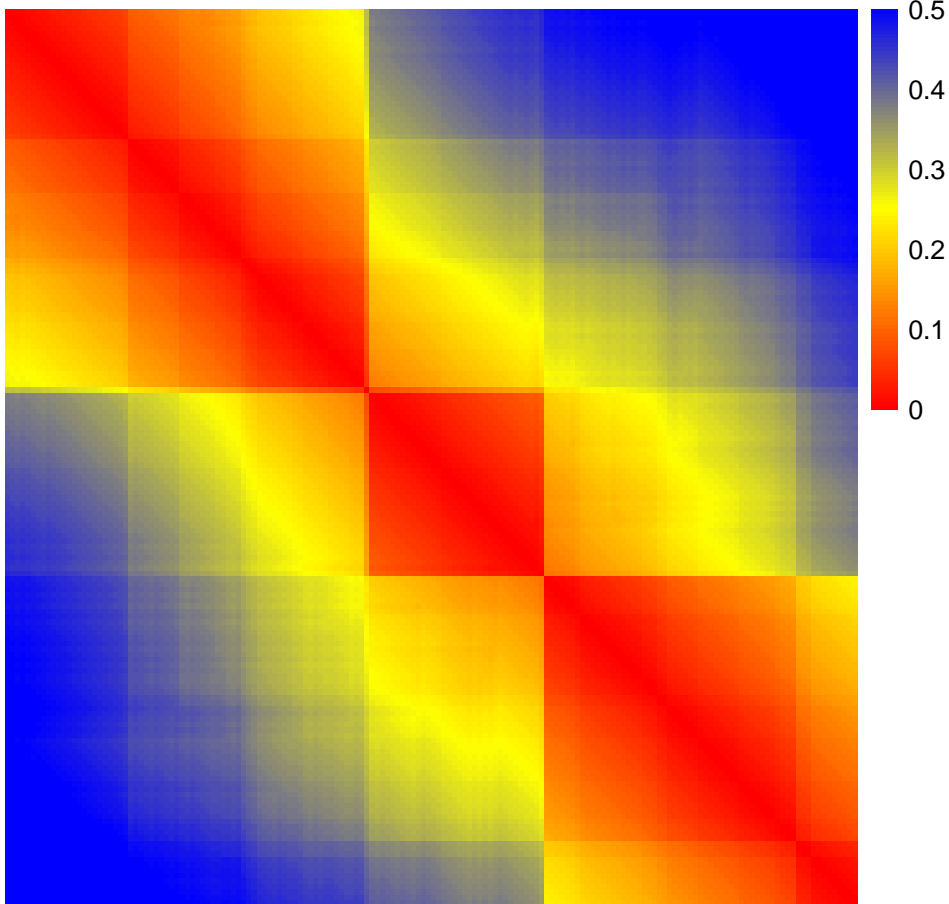

LG11

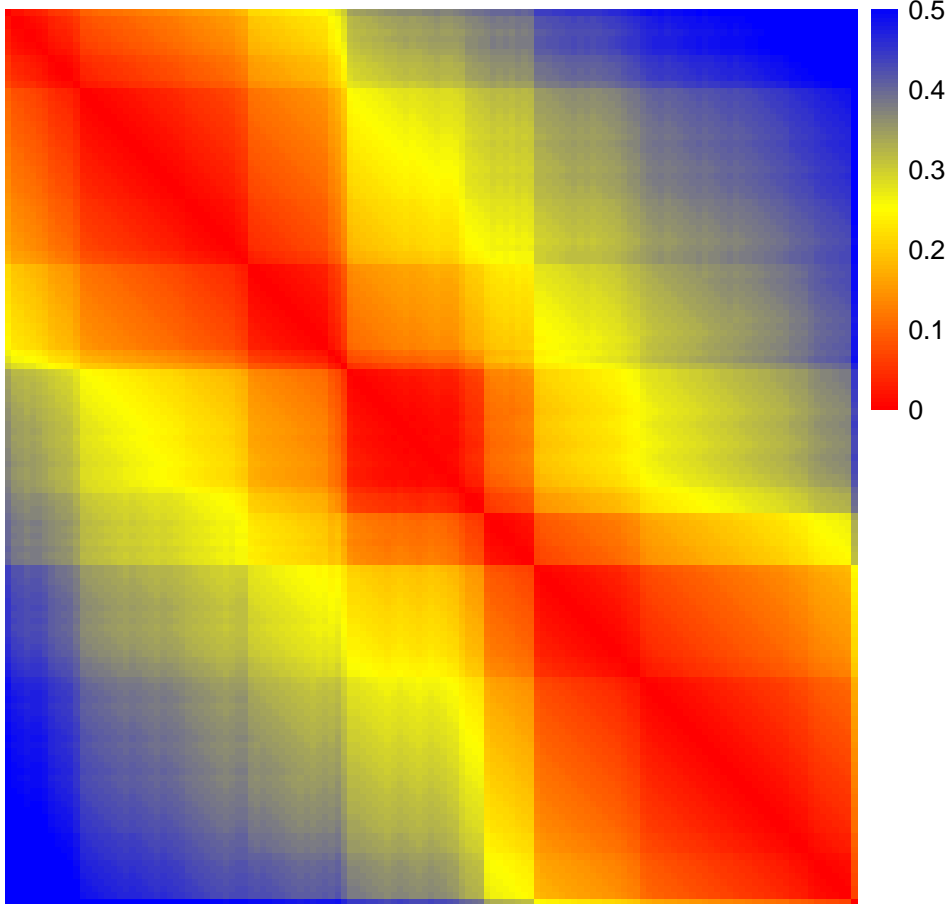

LG12

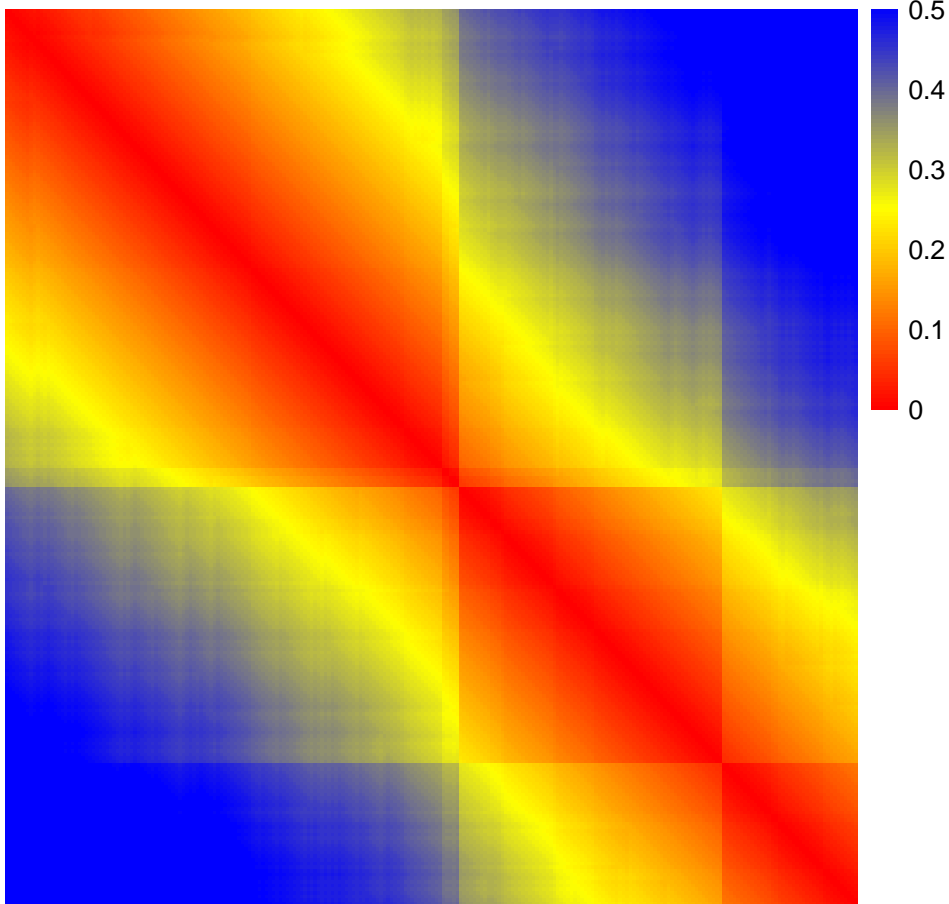

Supplement: Supplementary file 4 — Supplementary Material 4: Figure S1. Heatmaps showing the recombination frequencies among markers located on each linkage group to evaluate the quality of the genetic map using the pairwise recombination rate. The vertical and horizontal coordinates represented the markers located within the LG; a blue square indicated a high rate of recombination, while red squares indicated a low rate of recombination [file 12870_2024_5092_MOESM4_ESM.pdf]

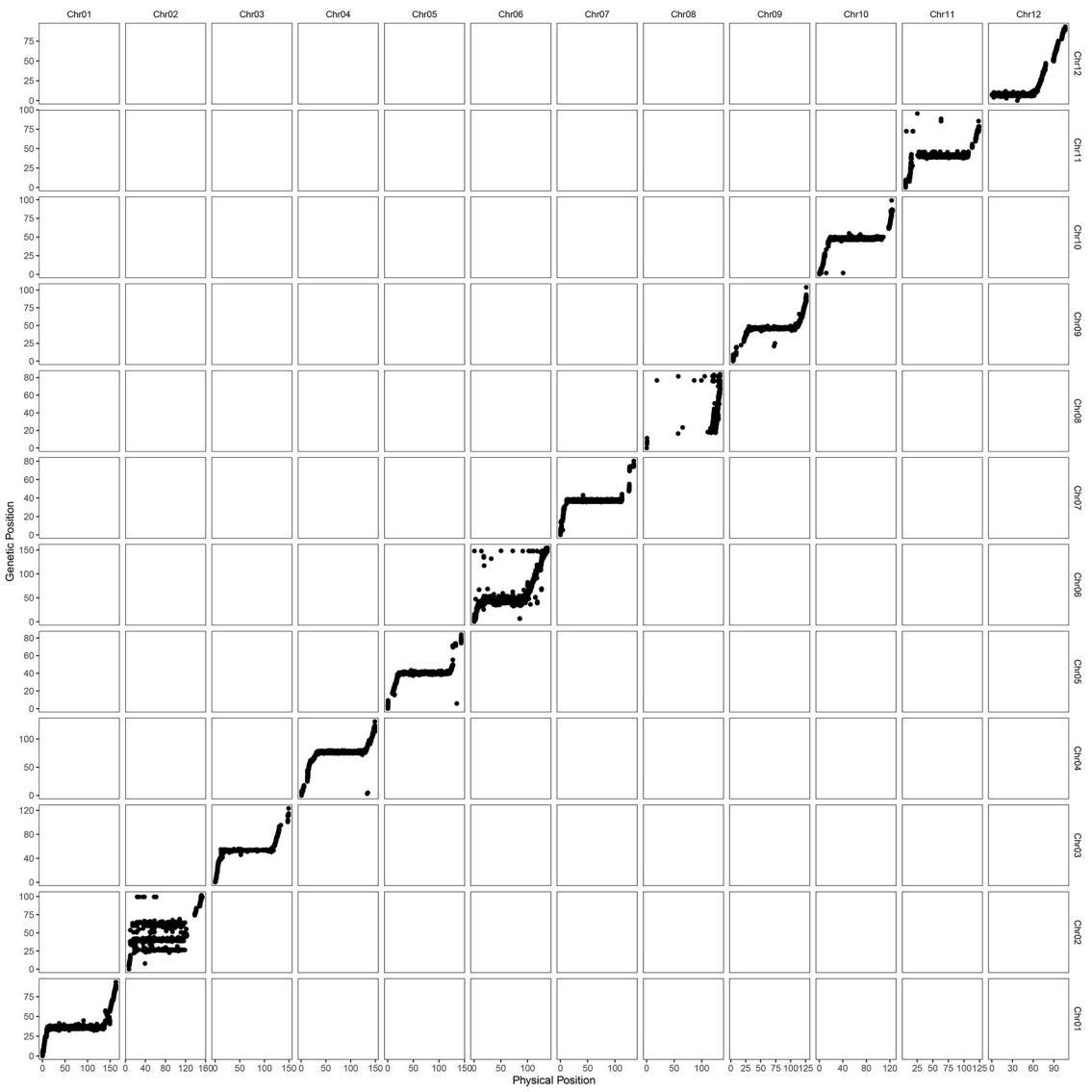

Supplement: Supplementary file 5 — Supplementary Material 5: Figure S2. Collinearity analysis of all goji linkage groups (LGs) with the goji genome sequence. The x-axis indicates the genetic distance of goji LGs; markers in these LGs are plotted as dots in the figure [file 12870_2024_5092_MOESM5_ESM.pdf]

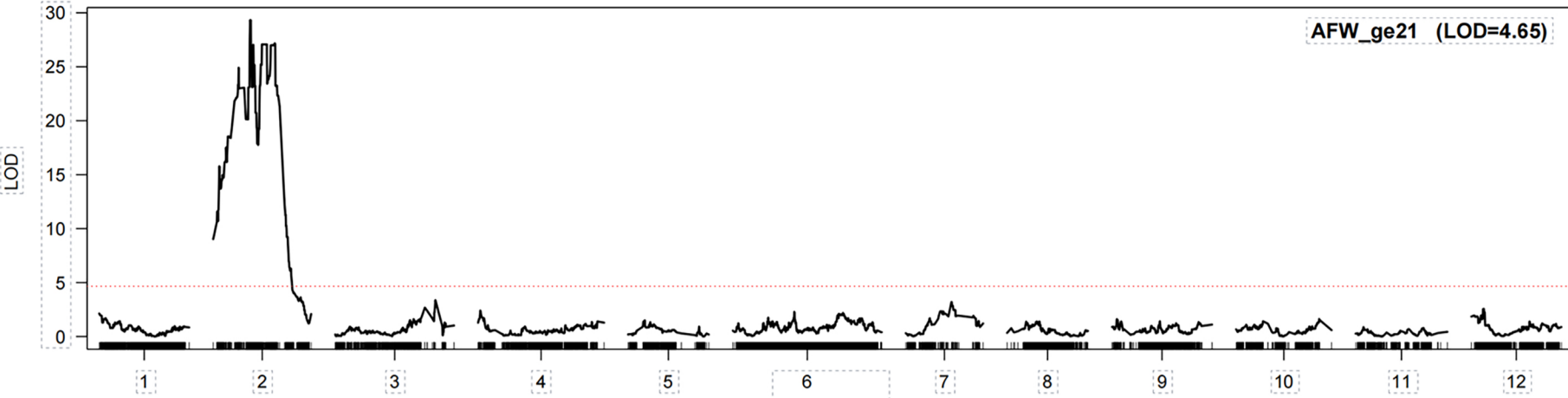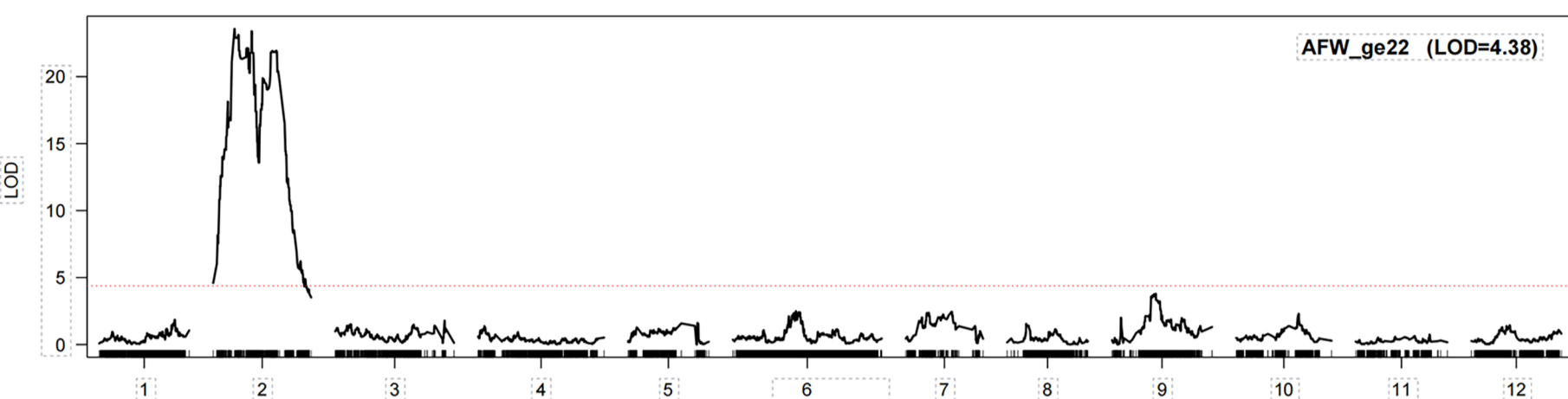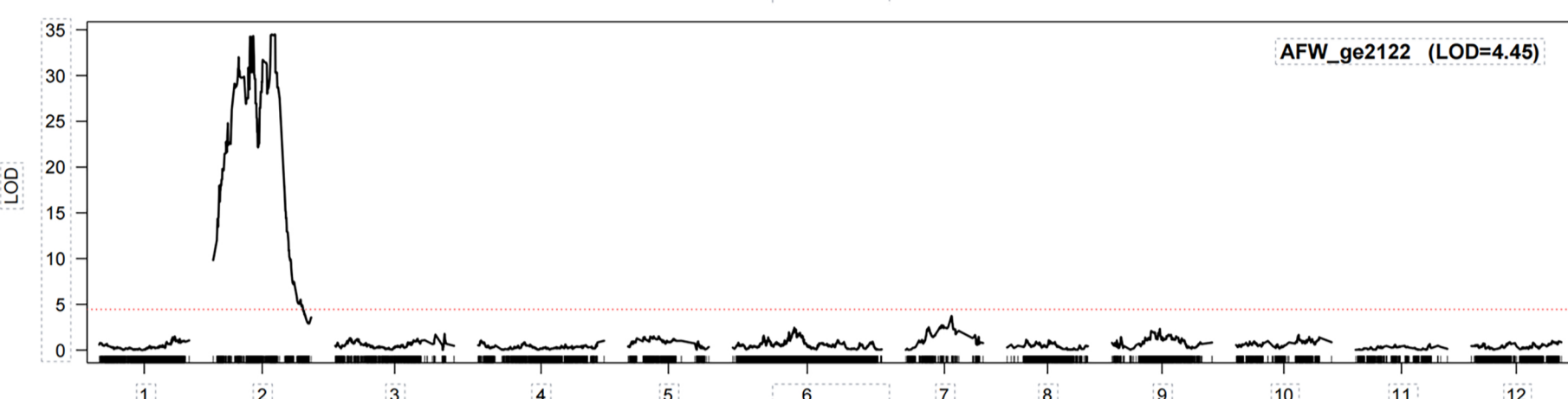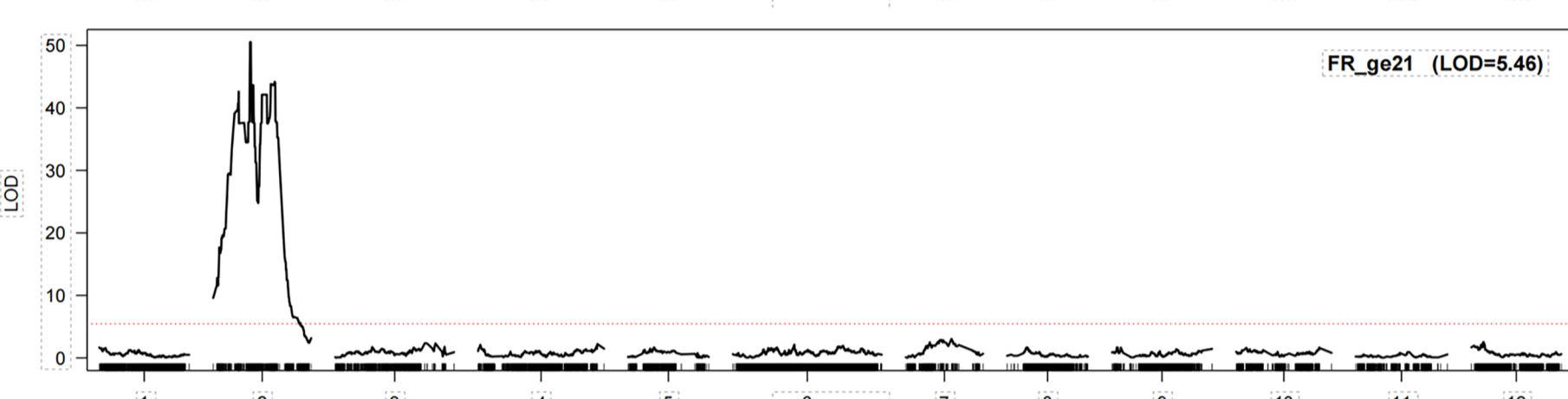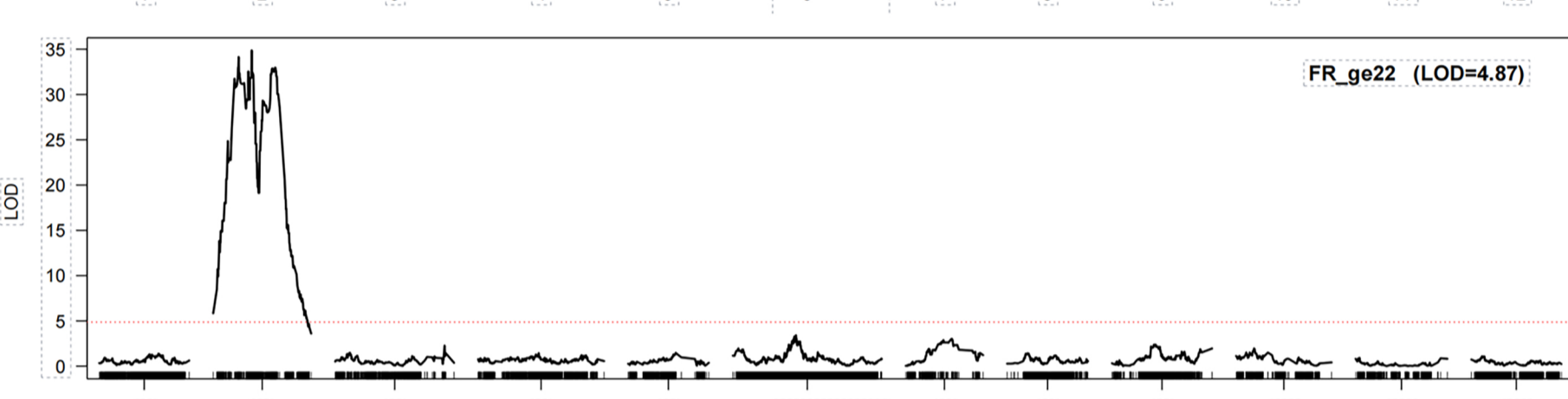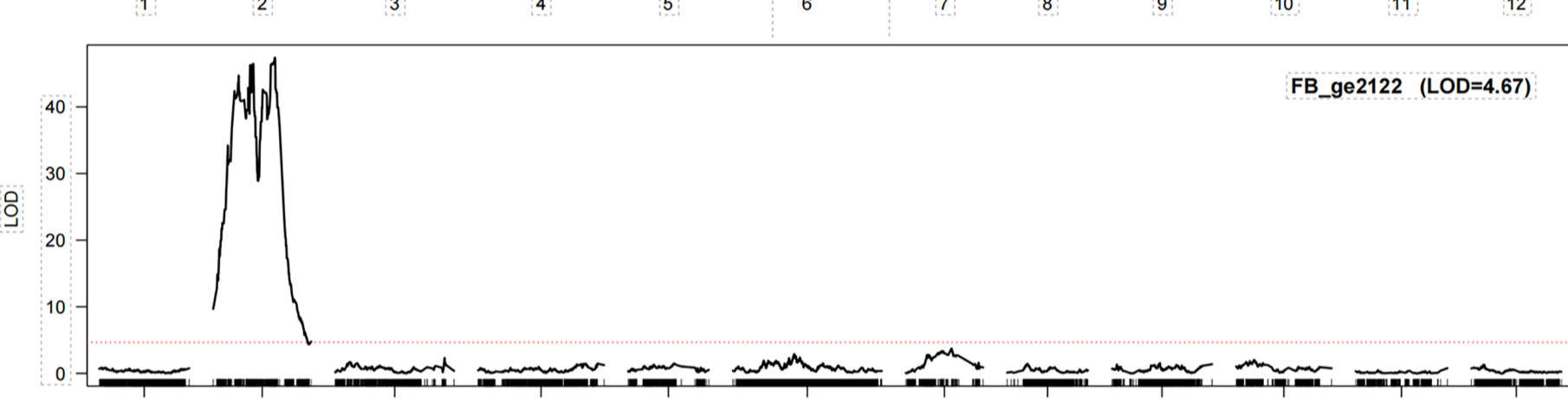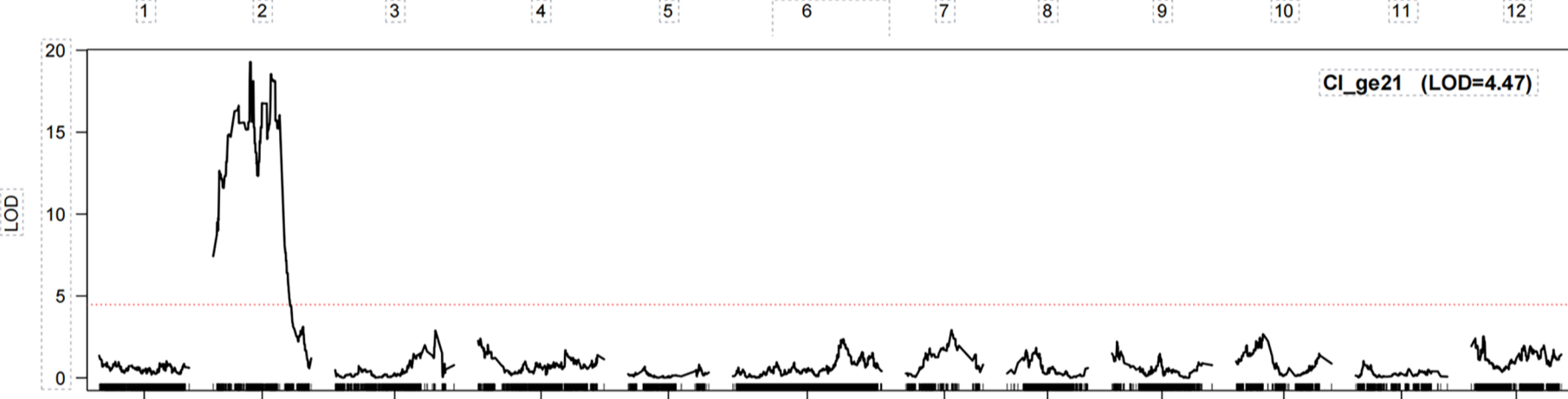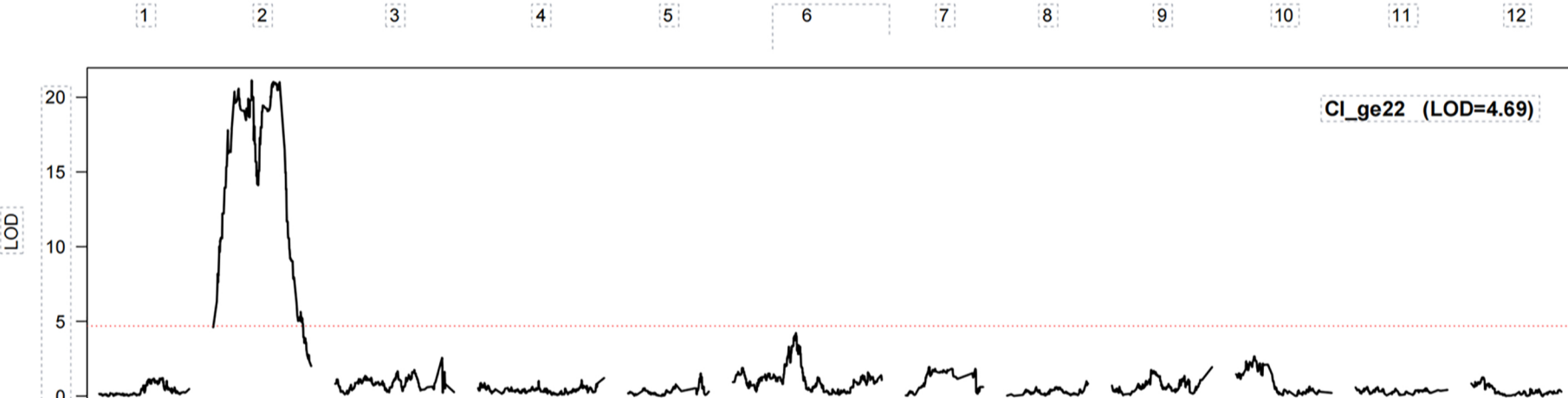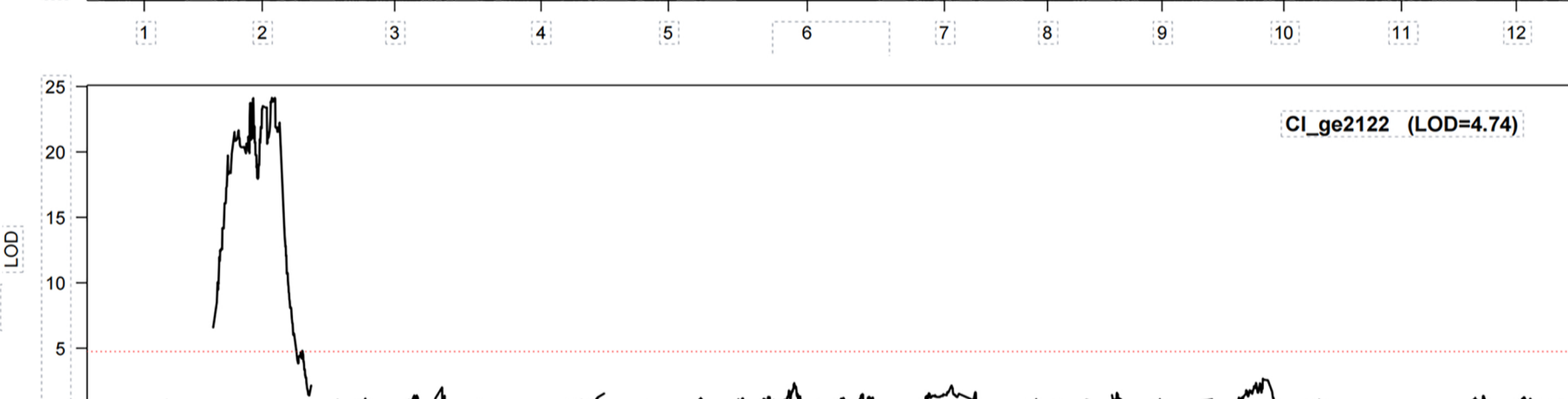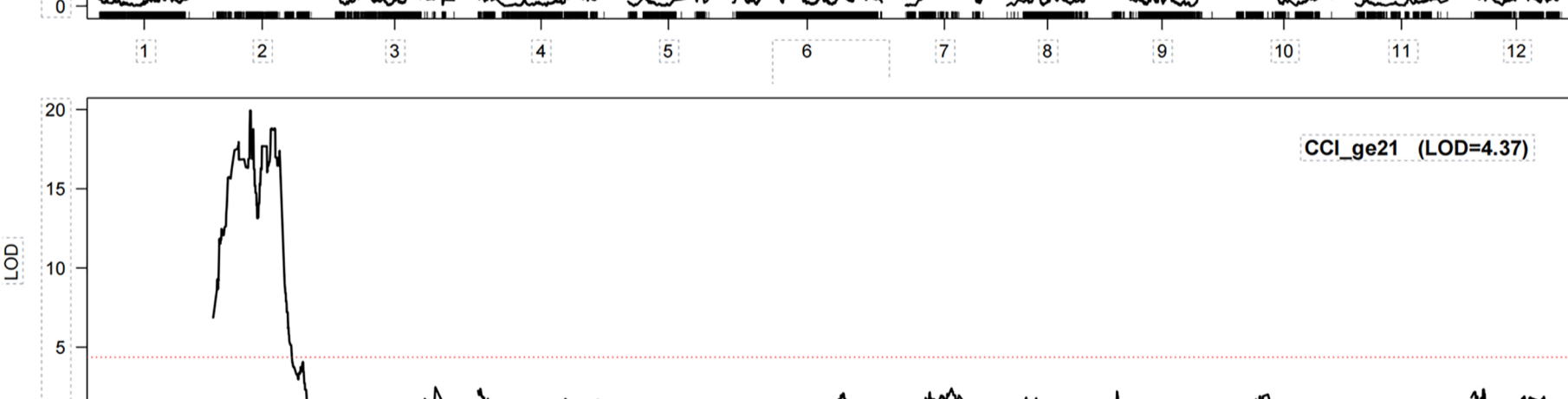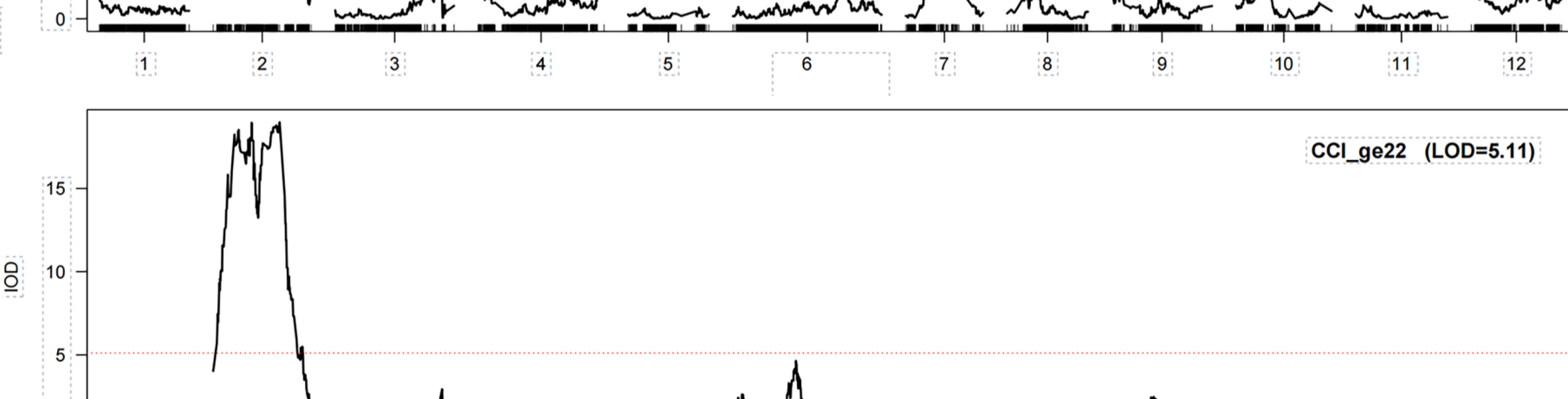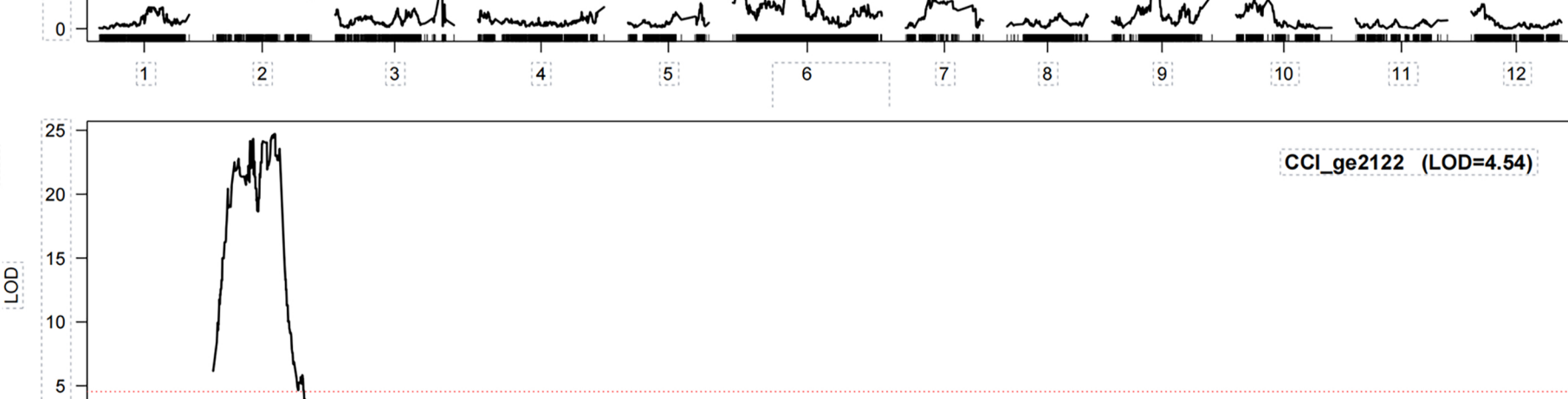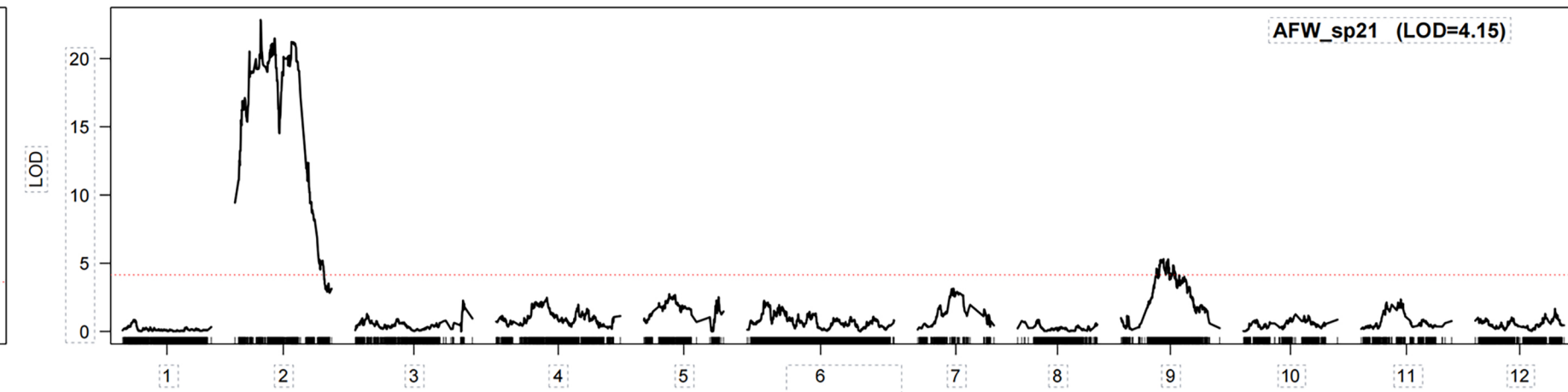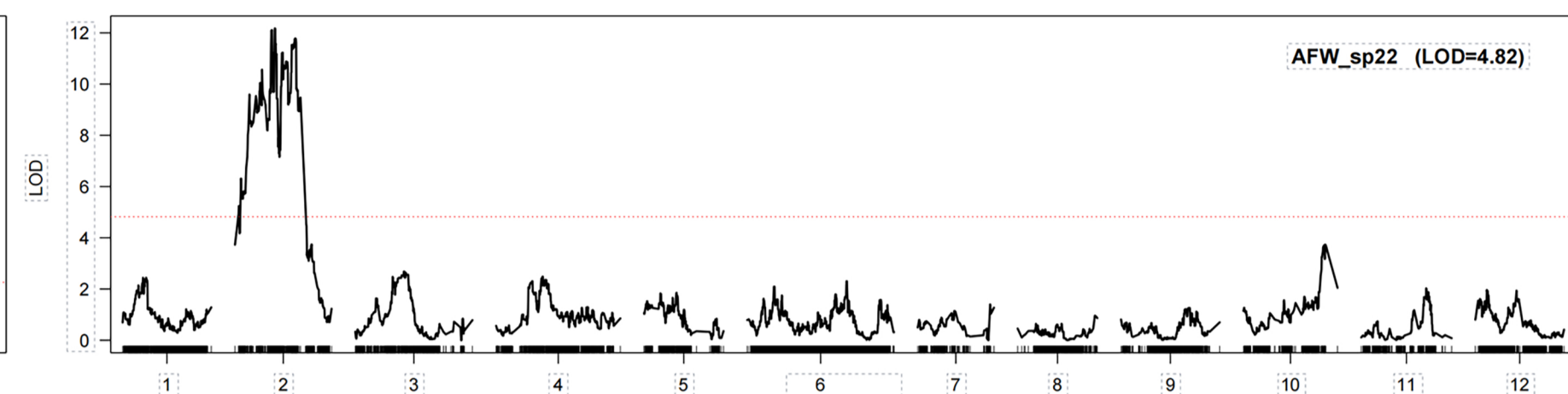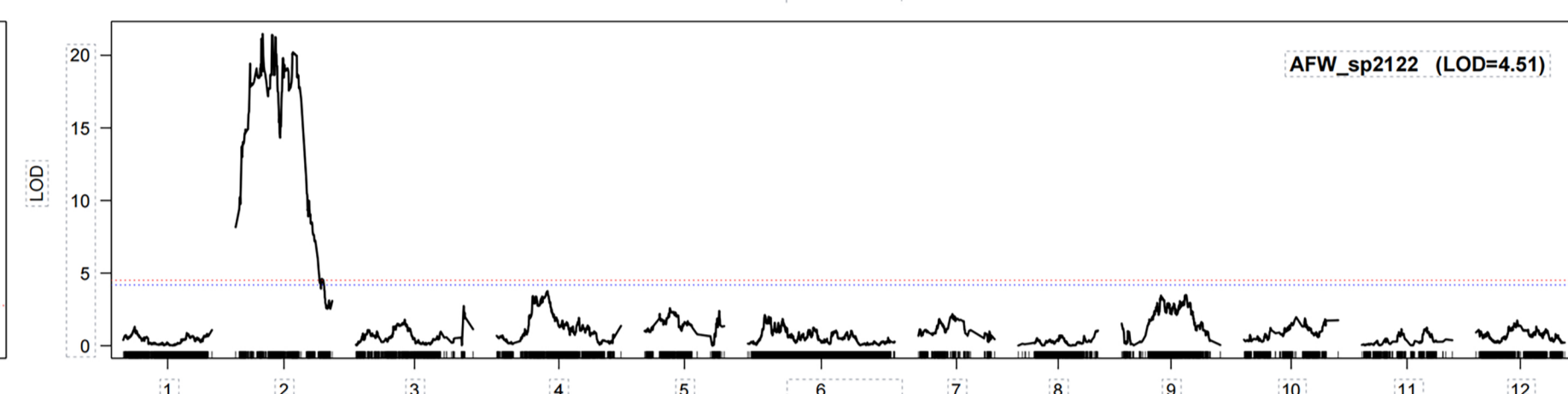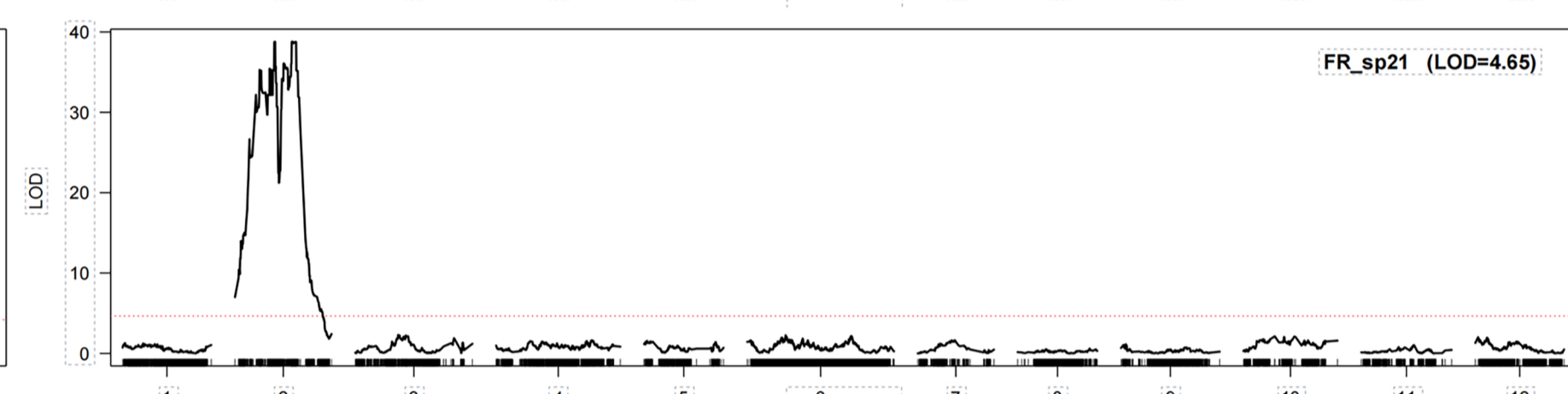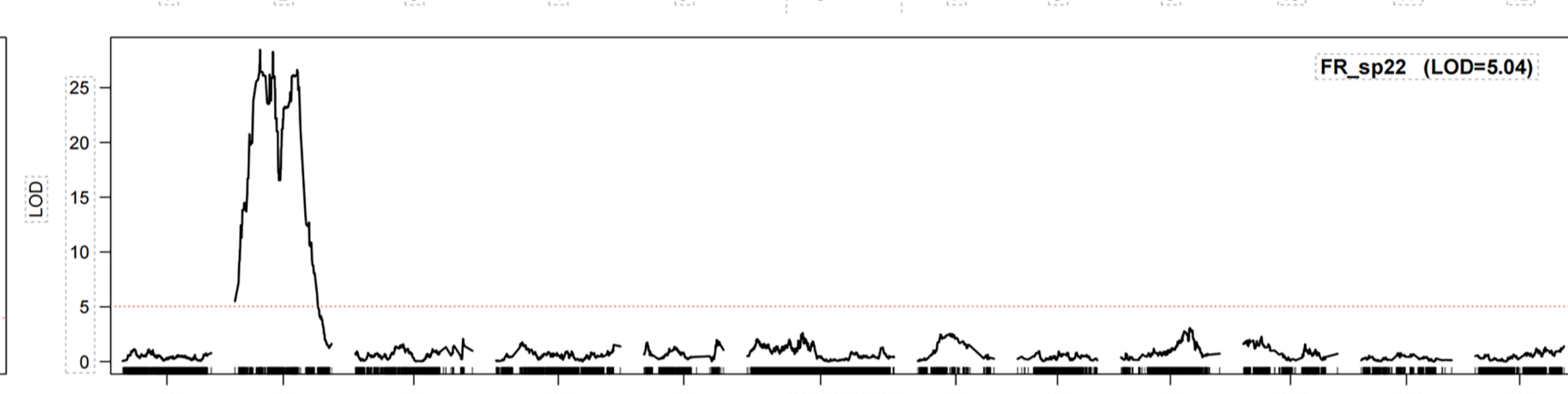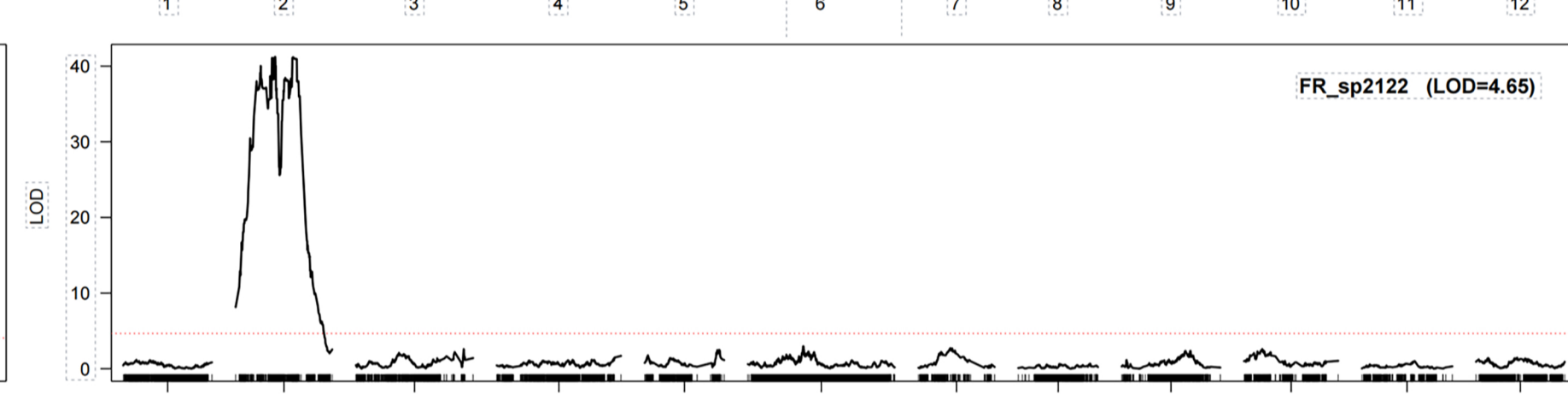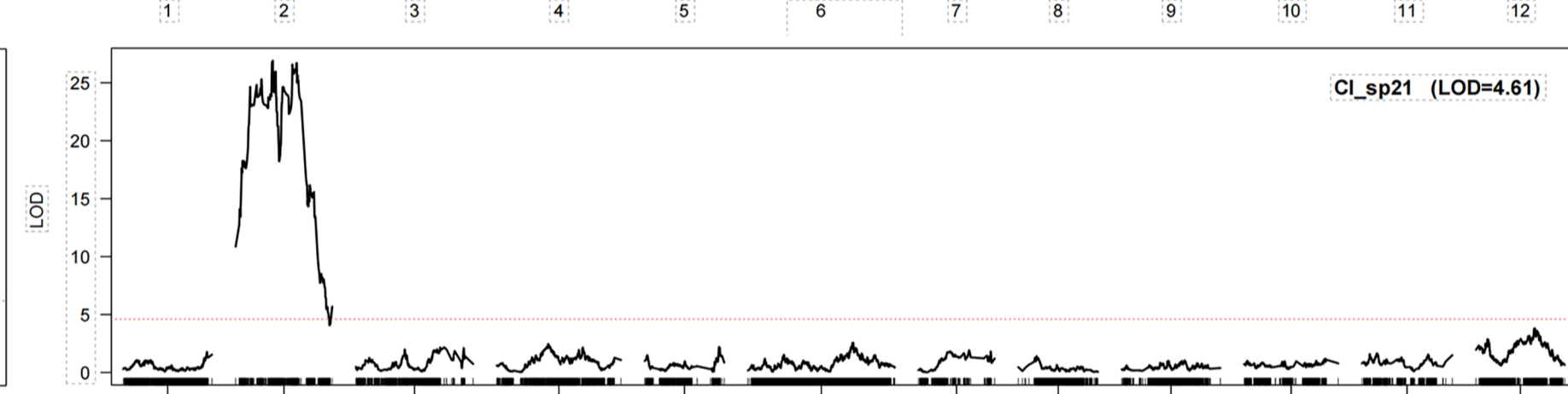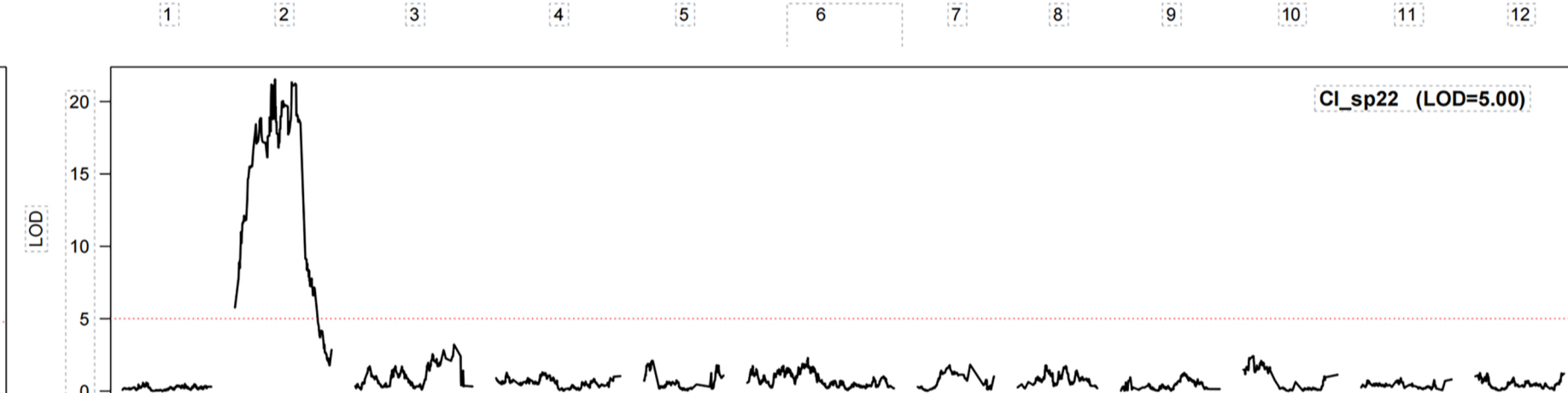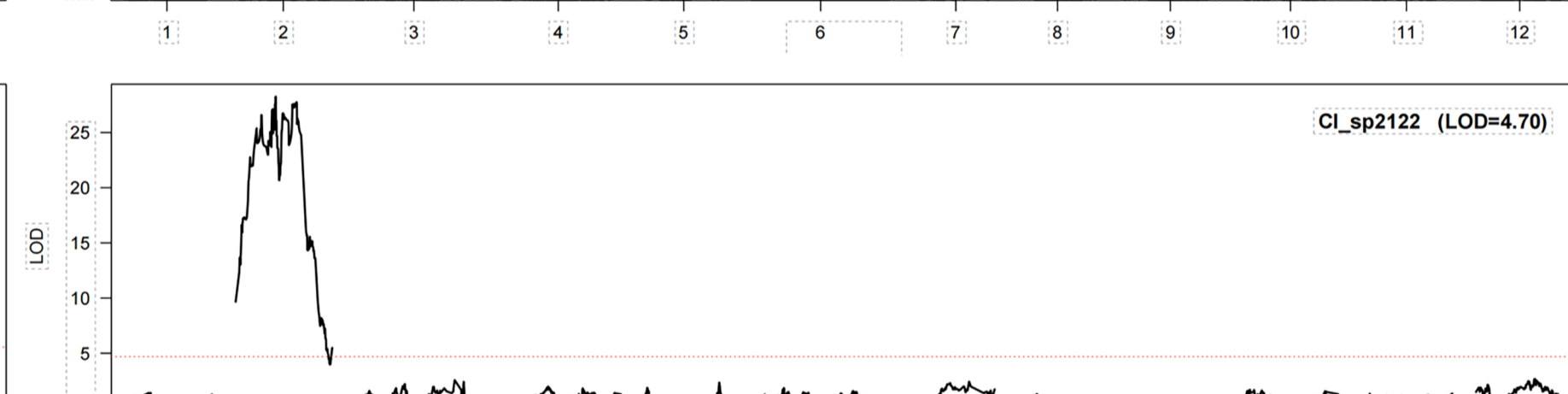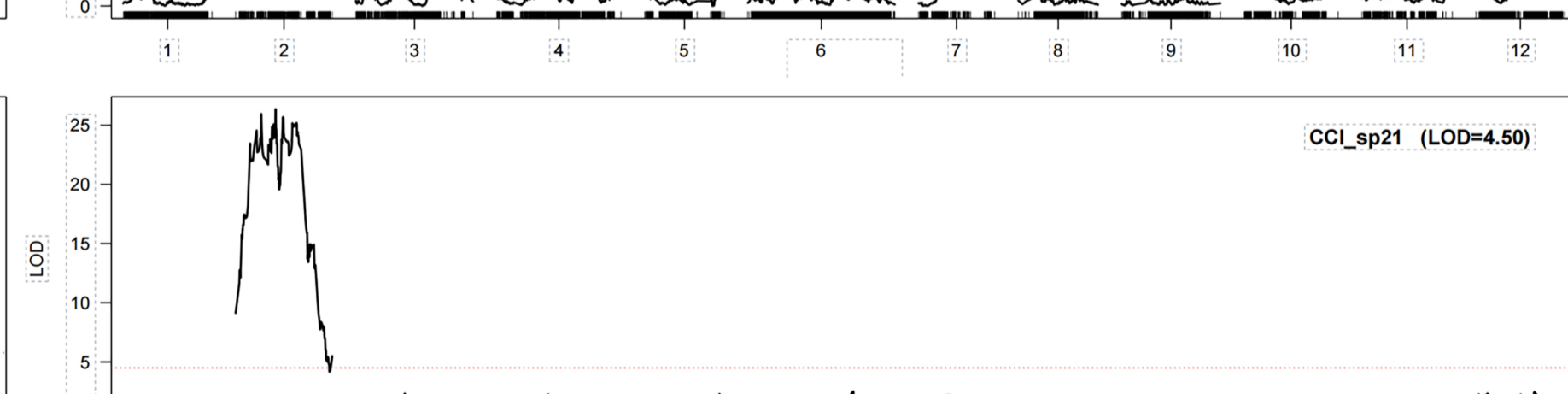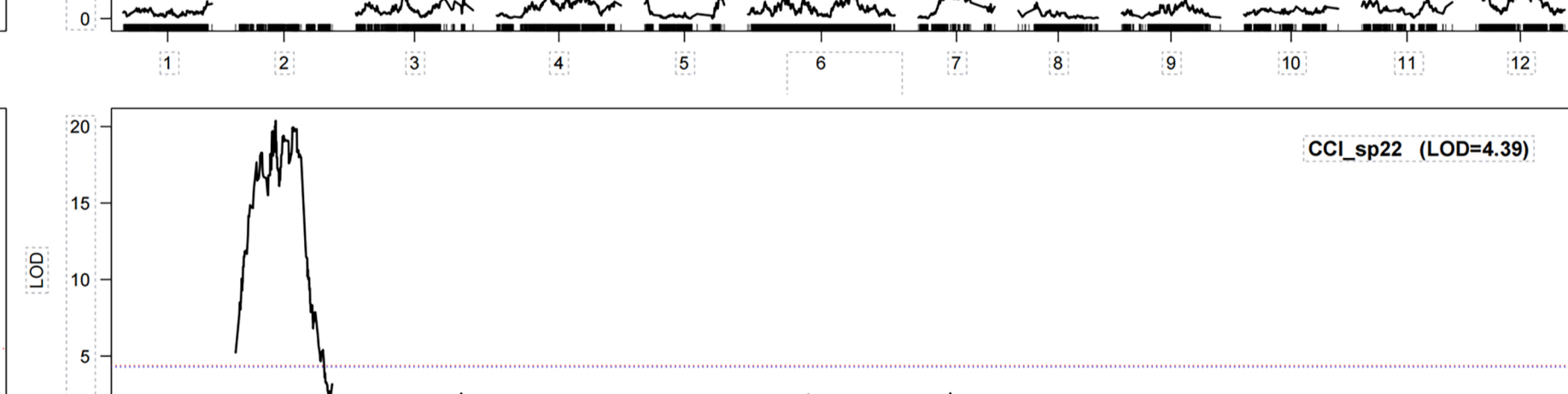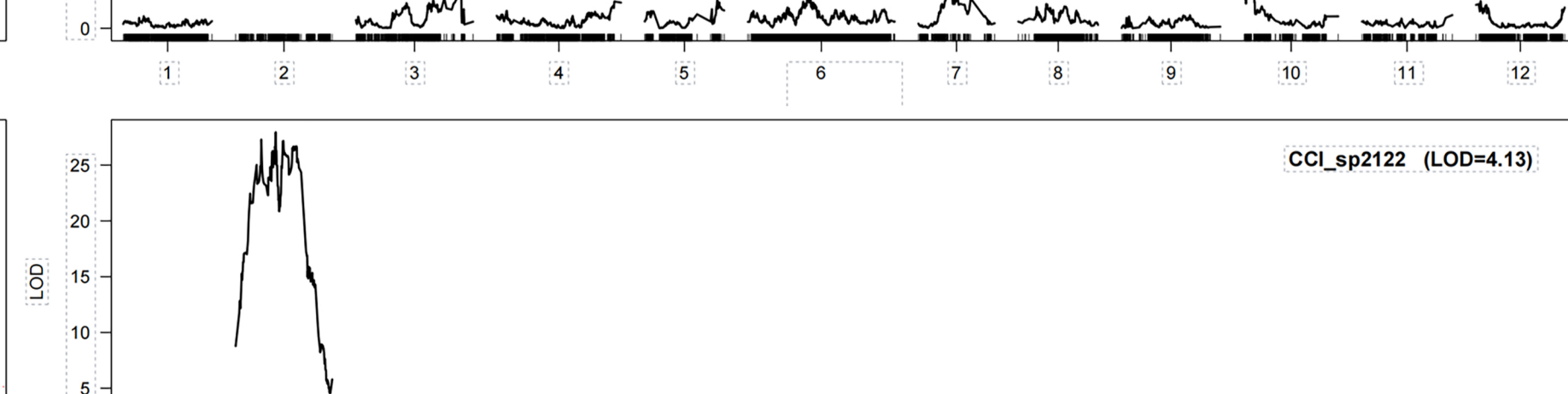

Supplement: Supplementary file 8 — Supplementary Material 8: Figure S3. QTL analysis for all investigated self-incompatibility related traits. FR, fruitful rate; AFW, average fruit weight; CI, self-compatibility index; CCI, compared compatibility index; _sp, collected after self-pollination; _ge, collected after geitonogamy [file 12870_2024_5092_MOESM8_ESM.pdf]
