# Supplementary material for: Mapping quantitative trait loci associated with self-(in)compatibility in goji berries (Lycium barbarum)
Source: BMC Plant Biol. 2024 May 23;24:441. doi: 10.1186/s12870-024-05092-7 (PMC11112781; doi:10.1186/s12870-024-05092-7)
Supplement: Supplementary file 7 — Supplementary Material 7: Table S5. Quantitative trait loci for self-incompatibility-related traits detected by composite interval mapping [file 12870_2024_5092_MOESM7_ESM.docx]

Table S5 Quantitative trait loci for self-incompatibility-related traits detected by composite interval mapping.

| Trait | Year | QTLs | LG | Peak cM | StartcM | EndcM | PeakMarker | StartMarker | EndMarker | LOD | PVE |
| --- | --- | --- | --- | --- | --- | --- | --- | --- | --- | --- | --- |
| FR_sp | 21 | qFRS2-1 | 2 | 60.365 | 0 | 92.285 | Chr02.107331126 | Chr02.7257846 | Chr02.149834098 | 38.79 | 78.28 |
|  | 22 | qFRS2-1.1 | 2 | 26.449 | 0 | 86.537 | Chr02.62339144 | Chr02.7257846 | Chr02.148530404 | 28.45 | 67.68 |
|  | 21/22 | qFRS2-1.2 | 2 | 41.685 | 0 | 92.860 | Chr02.22669022 | Chr02.7257846 | Chr02.150006699 | 41.21 | 71.54 |
| FR_ge | 21 | qFRG2-1 | 2 | 38.809 | 0 | 90.272 | Chr02.20560264 | Chr02.7257846 | Chr02.149366668 | 50.51 | 88.86 |
|  | 21 | qFRG2-1.1 | 2 | 90.847 | 90.847 | 90.847 | Chr02.149483252 | Chr02.149378913 | Chr02.149483252 | 5.57 | 21.50 |
|  | 22 | qFRG2-1.1 | 2 | 39.959 | 0 | 98.038 | Chr02.40713435 | Chr02.7257846 | Chr02.151794252 | 34.87 | 72.89 |
|  | 21/22 | qFRG2-1.2 | 2 | 64.101 | 0 | 98.032 | Chr02.103758491 | Chr02.7257846 | Chr02.151794252 | 47.38 | 78.26 |
|  | 21/22 | qFRG2-2 | 2 | 101.769 | 101.769 | 101.769 | Chr02.151796745 | Chr02.151796745 | Chr02.151796745 | 4.78 | 14.26 |
| AFW_sp | 21 | qAFWS2-1 | 2 | 27.025 | 0 | 93.722 | Chr02.75406258 | Chr02.7257846 | Chr02.150171108 | 22.83 | 48.60 |
|  | 21 | qAFWS9-1 | 9 | 37.681 | 37.106 | 38.544 | Chr09.25639681 | Chr09.25104012 | Chr09.26698752 | 4.60 | 12.55 |
|  | 21 | qAFWS9-2 | 9 | 44.866 | 40.268 | 50.621 | Chr09.31263094 | Chr09.27161108 | Chr09.112277495 | 5.30 | 14.31 |
|  | 21 | qAFWS9-3 | 9 | 52.345 | 52.057 | 52.345 | Chr09.112867906 | Chr09.112650763 | Chr09.113079352 | 4.25 | 11.65 |
|  | 21 | qAFWS9-4 | 9 | 54.931 | 54.069 | 56.080 | Chr09.114803909 | Chr09.114438630 | Chr09.116143308 | 4.84 | 13.16 |
|  | 21 | qAFWS9-5 | 9 | 56.655 | 56.655 | 56.655 | Chr09.116266874 | Chr09.116262447 | Chr09.116210365 | 4.38 | 11.98 |
|  | 21 | qAFWS9-6 | 9 | 57.517 | 57.517 | 57.517 | Chr09.116226458 | Chr09.116226458 | Chr09.116324474 | 4.22 | 11.57 |
|  | 22 | qAFWS2-1.1 | 2 | 4.030 | 3.743 | 4.317 | Chr02.7120869 | Chr02.7124275 | Chr02.7142211 | 5.24 | 19.53 |
|  | 22 | qAFWS2-1.2 | 2 | 41.976 | 5.4670 | 74.178 | Chr02.60393633 | Chr02.7378507 | Chr02.138592465 | 12.17 | 39.65 |
|  | 21/22 | qAFWS2-1.3 | 2 | 28.461 | 0 | 88.261 | Chr02.49869932 | Chr02.7257846 | Chr02.148336707 | 21.47 | 44.48 |
|  | 21/22 | qAFWS2-1.4 | 2 | 90.272 | 90.272 | 90.272 | Chr02.149311354 | Chr02.149273872 | Chr02.149366668 | 4.52 | 11.66 |
|  | 21/22 | qAFWS2-1.5 | 2 | 90.847 | 90.847 | 91.135 | Chr02.149483252 | Chr02.149378913 | Chr02.149305989 | 4.61 | 11.87 |
|  | 21/22 | qAFWS2-1.6 | 2 | 92.285 | 91.998 | 92.285 | Chr02.149817405 | Chr02.149683168 | Chr02.149834098 | 4.53 | 11.69 |
| AFW_ge | 21 | qAFWG2-1 | 2 | 38.809 | 0 | 81.649 | Chr02.18114759 | Chr02.7257846 | Chr02.141462165 | 29.31 | 72.01 |
|  | 22 | qAFWG2-1.1 | 2 | 22.138 | 0 | 94.296 | Chr02.17259660 | Chr02.7257846 | Chr02.150237262 | 23.56 | 60.45 |
|  | 22 | qAFWG2-1.2 | 2 | 95.446 | 95.158 | 96.595 | Chr02.150782125 | Chr02.150477055 | Chr02.150920564 | 4.89 | 17.49 |
|  | 21/22 | qAFWG2-1.3 | 2 | 63.814 | 0 | 93.147 | Chr02.38114827 | Chr02.7257846 | Chr02.150096055 | 34.50 | 67.85 |
| CI_sp | 21 | qCIS2-1 | 2 | 39.097 | 0 | 98.607 | Chr02.22071487 | Chr02.7257846 | Chr02.151950812 | 26.91 | 54.35 |
|  | 21 | qCIS2-2 | 2 | 101.769 | 101.769 | 101.769 | Chr02.151796745 | Chr02.151796745 | Chr02.151796745 | 5.68 | 15.25 |
|  | 22 | qCIS2-1.1 | 2 | 42.261 | 0 | 86.537 | Chr02.74580280 | Chr02.7257846 | Chr02.148530404 | 21.53 | 49.52 |
|  | 21/22 | qCIS2-1.2 | 2 | 42.261 | 0 | 97.745 | Chr02.74580280 | Chr02.7257846 | Chr02.151808962 | 28.24 | 54.12 |
|  | 21/22 | qCIS2-2.1 | 2 | 101.769 | 101.769 | 101.769 | Chr02.151796745 | Chr02.151796745 | Chr02.151796745 | 5.48 | 14.04 |
| CI_ge | 21 | qCIG2-1 | 2 | 38.522 | 0 | 79.350 | Chr02.50225033 | Chr02.7257846 | Chr02.140523861 | 19.28 | 56.72 |
|  | 22 | qCIG2-1.1 | 2 | 39.959 | 3.743 | 93.147 | Chr02.40713435 | Chr02.7124275 | Chr02.150096055 | 21.12 | 54.65 |
|  | 21/22 | qCIG2-1.3 | 2 | 60.940 | 0 | 83.948 | Chr02.68591991 | Chr02.7257846 | Chr02.141399860 | 24.13 | 54.02 |
|  | 21/22 | qCIG2-3 | 2 | 92.572 | 92.572 | 92.572 | Chr02.149872246 | Chr02.149873980 | Chr02.149866320 | 4.80 | 14.32 |
| CCI_sp | 21 | qCCIS2-1 | 2 | 41.973 | 0 | 98.607 | Chr02.60393633 | Chr02.7257846 | Chr02.151950812 | 26.35 | 52.28 |
|  | 21 | qCCIS2-2 | 2 | 101.769 | 101.769 | 101.769 | Chr02.151796745 | Chr02.151796745 | Chr02.151796745 | 5.49 | 14.28 |
|  | 22 | qCCIS2-1.1 | 2 | 42.261 | 0 | 93.147 | Chr02.74580280 | Chr02.7257846 | Chr02.150096055 | 20.38 | 47.88 |
|  | 21/22 | qCCIS2-1.2 | 2 | 42.261 | 0 | 101.769 | Chr02.74580280 | Chr02.7257846 | Chr02.151796745 | 27.94 | 53.09 |
| CCI_ge | 21 | qCCIG2-1 | 2 | 38.522 | 0 | 81.649 | Chr02.50225033 | Chr02.7257846 | Chr02.141462165 | 19.93 | 57.92 |
|  | 22 | qCCIG2-1.1 | 2 | 68.987 | 3.743 | 87.399 | Chr02.108219767 | Chr02.7124275 | Chr02.148826208 | 18.97 | 50.01 |
|  | 22 | qCCIG2-2 | 2 | 90.847 | 90.847 | 91.135 | Chr02.149483252 | Chr02.149378913 | Chr02.149305989 | 5.42 | 17.97 |
|  | 22 | qCCIG2-3 | 2 | 92.572 | 92.572 | 93.147 | Chr02.149872246 | Chr02.149873980 | Chr02.150096055 | 5.48 | 18.17 |
|  | 21/22 | qCCIG2-1.2 | 2 | 64.101 | 0 | 94.009 | Chr02.49348784 | Chr02.7257846 | Chr02.150099867 | 24.72 | 54.88 |

FR, fruitful rate; AFW, average fruit weight; CI, self-compatibility index; CCI, compared compatibility index; _sp, collected after self-pollination; _ge, collected after geitonogamy
