# Supplementary material for: Mapping quantitative trait loci associated with self-(in)compatibility in goji berries (Lycium barbarum)
Source: BMC Plant Biol. 2024 May 23;24:441. doi: 10.1186/s12870-024-05092-7 (PMC11112781; doi:10.1186/s12870-024-05092-7)
Supplement: Supplementary file 10 — Supplementary Material 10: Figure S4-S8. PCR Amplification electrophoresis of S genotype in parents and F1 population [file 12870_2024_5092_MOESM10_ESM.docx]

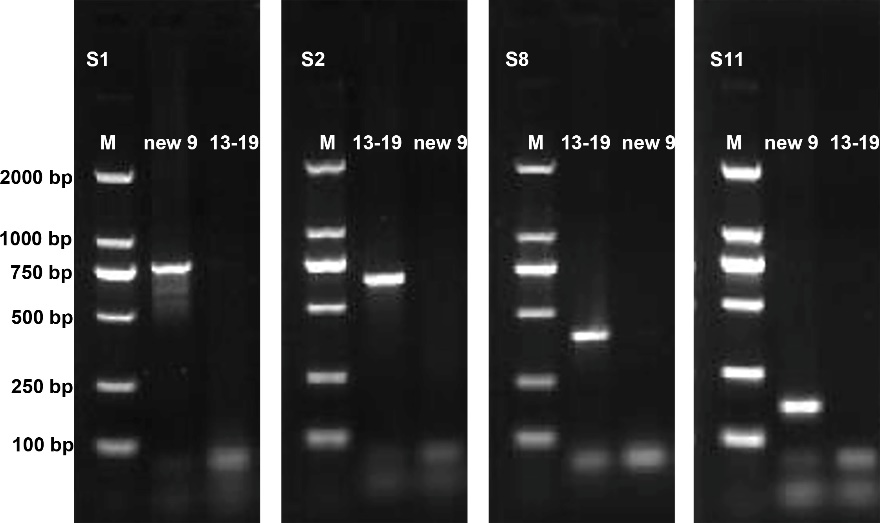


Figure S4 Gel electrophoresis bands of different S gene type identification primers in the parents. M, molecular weight marker; ‘new 9’ and ‘13-19’ indicate two different parents. The band sizes are S1 810 bp, S2 666 bp, S8 400 bp, S11 169 bp.


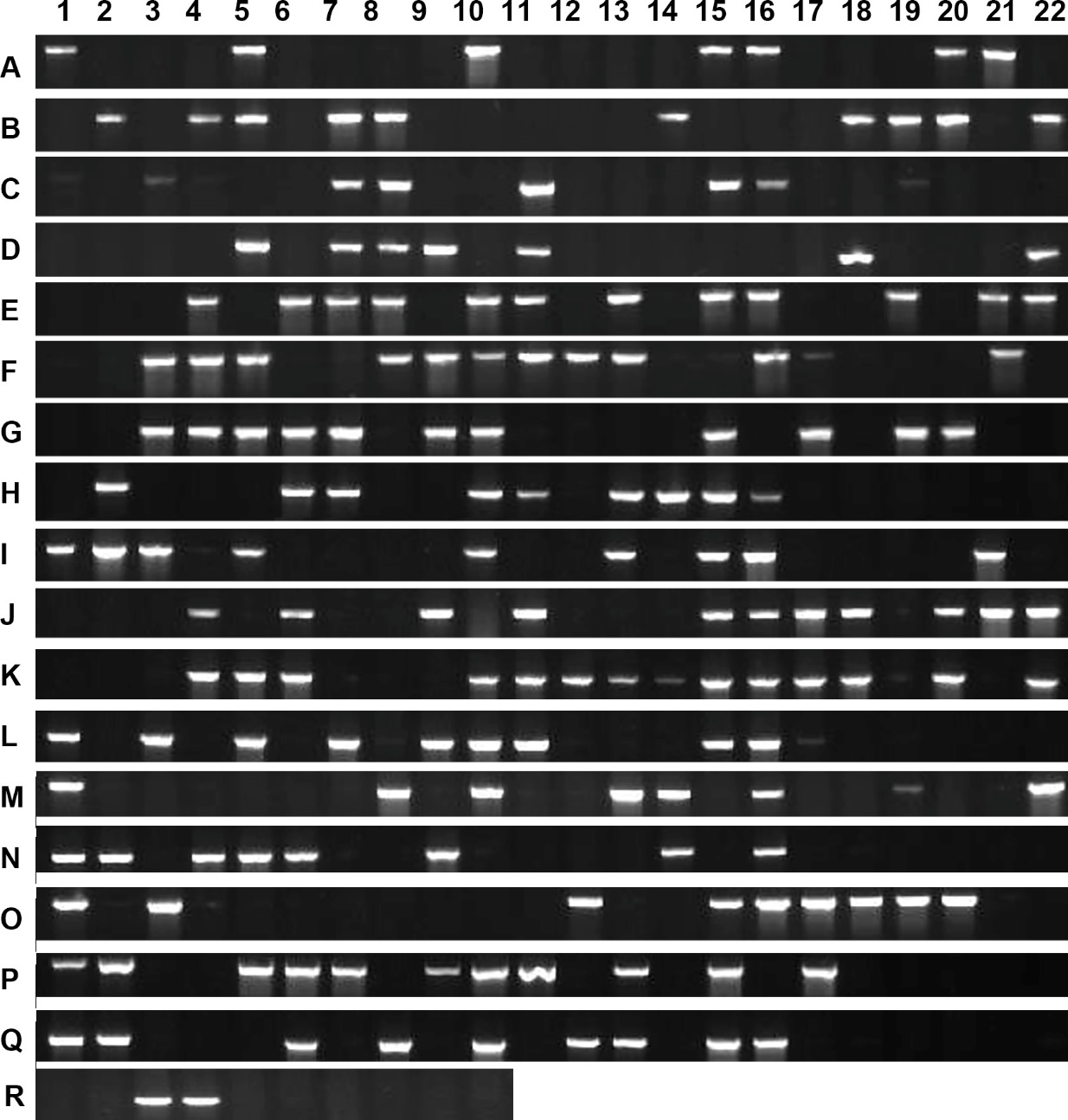


Figure S5 Amplification electrophoresis bands of S2 genotype in each individual of the F1 population.


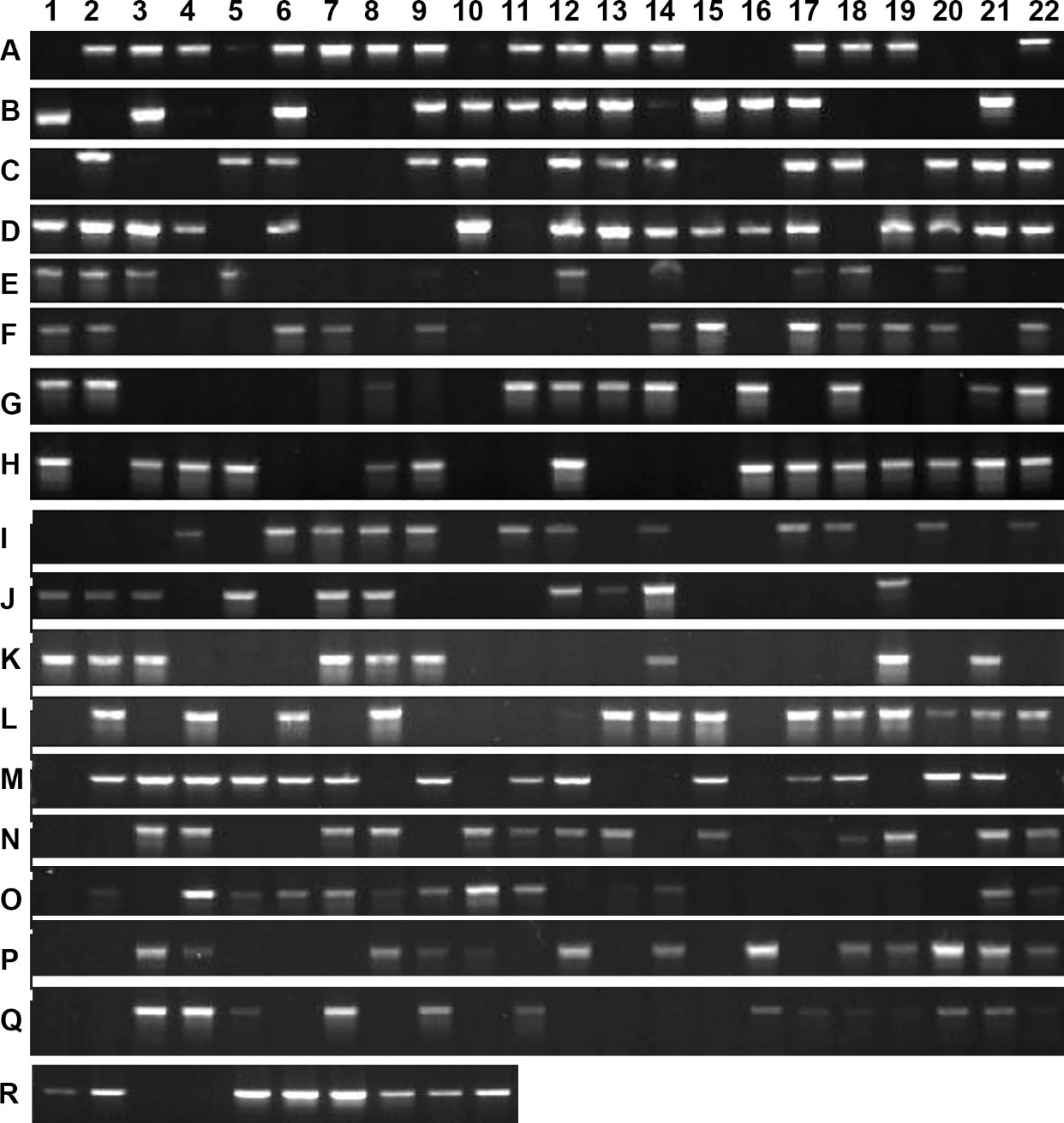


Figure S6 Amplification electrophoresis bands of S8 genotype in each individual of the F1 population.


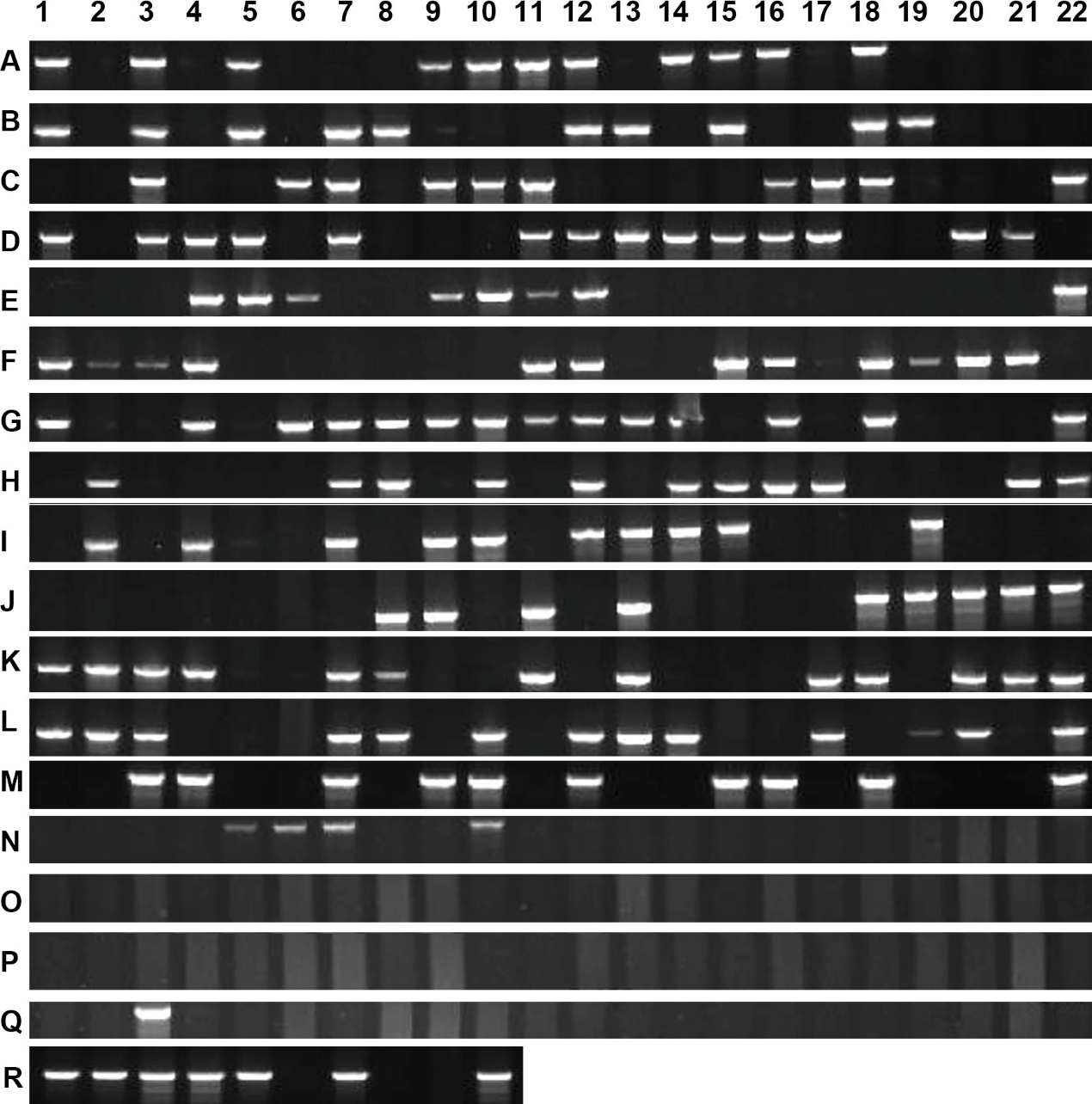


Figure S7 Amplification electrophoresis bands of S1 genotype in each individual of the F1 population.


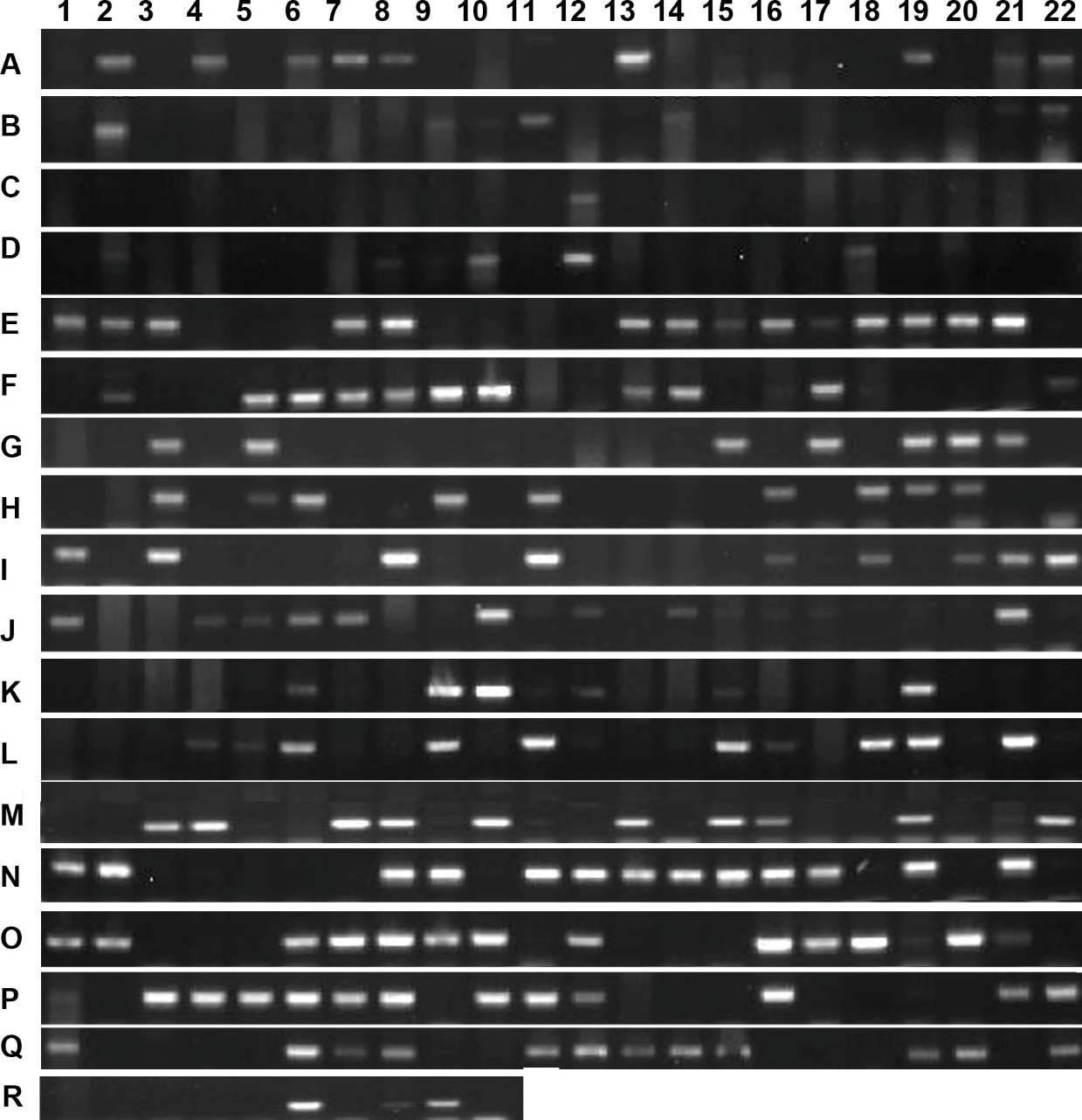


Figure S8 Amplification electrophoresis bands of S11 genotype in each individual of the F1 population.
